# Targeting the RNF31–TFEB–NLRP3 Axis With a Curcumin Analog to Restore Autophagy and Alleviate Intestinal Inflammation

**DOI:** 10.1002/fsn3.71916

**Published:** 2026-05-23

**Authors:** Lu Han, Yang Xie, Chunyan Zeng, Youxiang Chen

**Affiliations:** ^1^ Department of Gastroenterology, Jiangxi Provincial Key Laboratory of Digestive Diseases, Jiangxi Clinical Research Center for Gastroenterology, Digestive Disease Hospital The First Affiliated Hospital, Jiangxi Medical College Nanchang University Nanchang Jiangxi China; ^2^ Postdoctoral Research Station, The First Affiliated Hospital, Jiangxi Medical College Nanchang University Nanchang People's Republic of China; ^3^ Digestive Diseases Center, Guangdong Provincial Key Laboratory of Digestive Cancer Research The Seventh Affiliated Hospital, Sun Yat‐Sen University Shenzhen Guangdong People's Republic of China; ^4^ Department of Gastroenterology Jiangxi Province Hospital of Integrated Chinese and Western Medicine Nanchang Jiangxi China

**Keywords:** autophagy dysfunction, CM‐C1, inflammatory bowel disease, RNF31, TFEB

## Abstract

Inflammatory bowel disease (IBD) is characterized by impaired autophagy and chronic inflammation. Although the E3 ubiquitin ligase RNF31 is upregulated in IBD, its pathogenic mechanisms remain incompletely understood. To address this, a combination of in vitro and in vivo methods was employed. In vitro, lipopolysaccharide (LPS)‐stimulated cell models were used to analyze transcription factor EB (TFEB) phosphorylation, its interaction with RNF31, ubiquitination, and subcellular localization. In vivo, a DSS‐induced IBD mouse model was used to assess intestinal pathology, inflammation, and RNF31‐TFEB‐NLRP3 axis proteins after treatment with a novel synthetic curcumin analog (CM‐C1). We identified TFEB as a novel substrate of RNF31. LPS‐induced phosphorylation of TFEB promoted its binding to RNF31 (via TFEB‐S281/T276 and RNF31‐K908), leading to TFEB ubiquitination, proteasomal degradation, suppressed autophagy, and subsequent NLRP3 inflammasome activation. The bioavailable TFEB activator CM‐C1 directly disrupted the RNF31‐TFEB interaction. This action promoted TFEB nuclear translocation, restored autophagic flux, alleviated intestinal inflammation in vitro and in vivo, and beneficially remodeled the gut microbiota. Our study unveils the RNF31‐TFEB‐NLRP3 axis as a pivotal pathogenic pathway in IBD and nominates CM‐C1, which targets this axis, as a promising multimodal therapeutic candidate.

## Graphical abstract

Under stimulation with inflammatory inducers such as LPS, RNF31 expression is upregulated. Meanwhile, TFEB undergoes mTORC1‐mediated phosphorylation at Ser211, which promotes the interaction between the K908 site of RNF31 and the S281/T276 sites of TFEB, leading to TFEB degradation via the ubiquitin‐proteasome pathway. This process reduces intracellular TFEB levels, resulting in insufficient nuclear TFEB content, which subsequently activates the downstream NLRP3 inflammasome, upregulates the inflammatory cytokine IL‐18, disrupts intestinal barrier integrity, and inhibits autophagy. However, upon addition of CM‐C1, TFEB levels are significantly increased, promoting its extensive nuclear entry, thereby reducing the binding between TFEB and RNF31 and preventing its degradation by the ubiquitin‐proteasome system. Nuclear‐localized TFEB inhibits NLRP3 inflammasome activation, thus protecting intestinal barrier integrity and promoting autophagic formation.

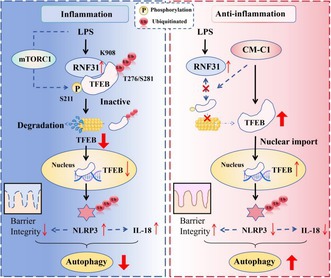

## 1 Introduction

Inflammatory bowel disease (IBD) is a chronic and relapsing disorder of the gastrointestinal tract characterized by persistent nonspecific intestinal inflammation, posing a significant challenge in clinical management. Current treatment strategies—including immunosuppressants, biologics, small‐molecule inhibitors, and surgery—are often limited by issues such as loss of response or post‐surgical recurrence (Gisbert and Chaparro 2024). Therefore, there is a pressing need to elucidate the molecular drivers of IBD and develop novel therapies that effectively suppress inflammation, restore the mucosal barrier, and promote tissue repair.

Accumulating evidence indicates that ring finger protein 31 (RNF31), a member of the RBR E3 ubiquitin ligase family, is significantly upregulated in patients with ulcerative colitis (UC) (Zhang, Tian, et al. 2024; Wang et al. 2023; Tang et al. 2024). *RNF31*‐knockout (*RNF31*‐KO) mice are protected from dextran sulfate sodium (DSS)‐induced colitis, underscoring its pro‐inflammatory role (Wang et al. 2023; Tang et al. 2024). RNF31 modulates inflammatory signaling by promoting the ubiquitination of specific substrates; however, its precise mechanisms in regulating the inflammatory cascade in IBD, particularly its connection to autophagy, remain unclear. Through bioinformatics screening, we identified transcription factor EB (TFEB), a master regulator of autophagy and lysosomal biogenesis, as an RNF31‐interacting protein with high potential relevance to IBD pathogenesis. Autophagy dysfunction has been widely implicated in the development and progression of IBD (Larabi et al. 2020; Subramanian et al. 2024), and impaired TFEB function has also been linked to IBD (Du et al. 2023; Jin et al. 2023). Nevertheless, whether and how RNF31 regulates autophagy via TFEB in the context of IBD remains unknown. Deciphering how the RNF31‐TFEB axis regulates autophagic flux may reveal novel therapeutic targets for IBD.

The natural compound curcumin possesses anti‐inflammatory and antioxidant activities and can alleviate intestinal inflammation by modulating autophagy in epithelial cells (Du et al. 2023; Cai, Liang, et al. 2024). However, its clinical translation is limited by poor aqueous solubility, low oral bioavailability, and rapid metabolism. A novel synthetic curcumin analog, CM‐C1, was rationally designed to overcome the pharmacokinetic limitations of curcumin. Key structural modifications, such as the removal of hydrogen‐bonding phenolic groups and the introduction of lipophilic substituents, reduce its molecular polarity and increase membrane permeability. Additionally, the incorporation of stable chemical moieties protects it from rapid hydrolysis and metabolic degradation (e.g., by glucuronidation). These optimizations collectively enhance its lipophilicity, metabolic stability, and gastrointestinal absorption, resulting in significantly improved oral bioavailability compared to the parent compound (Genchi et al. 2024; Song et al. 2016; Laurindo et al. 2023; Mohammadzadeh et al. 2024). CM‐C1 has been identified as a novel TFEB activator that promotes TFEB nuclear translocation and enhances autophagic flux without affecting its phosphorylation status (He et al. 2023; Zhuang et al. 2020). Although some reports suggest that CM‐C1 may exert protective effects by modulating autophagy (He et al. 2025; Jiang et al. 2023; Chen et al. 2024), its specific mechanism of action in gastrointestinal diseases remains unclear, and most related research is still in its early stages (Genchi et al. 2024; Song et al. 2016). Our preliminary data confirm that CM‐C1 effectively attenuates intestinal inflammation in DSS‐induced IBD mice, prompting us to investigate the molecular mechanisms by which it disrupts inflammatory signaling and rescues autophagy.

Classic studies have established that activated autophagy can restrain excessive NLRP3 inflammasome activation through multiple mechanisms. Notably, selective autophagy receptors such as p62/SQSTM1 can recognize ubiquitinated NLRP3 inflammasome components and target them for autophagic degradation. For instance, IRGM directly interacts with NLRP3 and ASC and mediates their SQSTM1/p62‐dependent autophagic degradation (Mehto et al. 2019). The E3 ubiquitin ligase TRIM20 ubiquitinates NLRP3, NLRP1 and caspase‐1, thereby initiating their p62‐dependent autophagic clearance (Hennig et al. 2021). Conversely, impairment of autophagic flux leads to accumulation of NLRP3 inflammasome components and exacerbates inflammatory responses in the intestine (Jiang et al. 2025). These findings collectively support that autophagy serves as a critical brake on NLRP3 inflammasome activation. However, whether and how the RNF31‐TFEB axis modulates this autophagic checkpoint remains unknown.

Lipopolysaccharide (LPS) stimulation in human colonic epithelial cells (NCM460) upregulates RNF31 expression and enhances TFEB phosphorylation. We further demonstrate that phosphorylated TFEB interacts with RNF31, leading to TFEB ubiquitination and proteasomal degradation. The degradation of TFEB reduces its nuclear translocation, suppresses autophagic flux, and ultimately exacerbates the inflammatory response (Wang, Wang, et al. 2024; Zhang et al. 2023; Cai, Liang, et al. 2024). Conversely, CM‐C1 effectively blocks the RNF31‐TFEB interaction, promotes TFEB nuclear localization, and exerts intestinal anti‐inflammatory effects by restoring autophagic flux. Importantly, we identified S281 and T276 residues of TFEB as critical sites for its binding to RNF31 and subsequent degradation. Using both in vitro *and* in vivo models of colitis, we verified that CM‐C1 counteracts the pro‐inflammatory effects of LPS by inhibiting the RNF31‐TFEB‐NLRP3 pathway. Our findings not only uncover a novel signaling axis driving intestinal inflammation but also highlight the considerable potential of CM‐C1 as a multi‐target therapeutic agent for IBD.

## 2 Materials and Methods

### 2.1 Cell Culture, Plasmids, and Antibodies

HEK293T, NCM460 cells were obtained from the Cancer Research Institute of Central South University and authenticated by short tandem repeat profiling. HEK293T were maintained in Dulbecco's Modified Eagle Medium (DMEM), while NCM460 cells were cultured in RPMI‐1640 medium; both media were supplemented with 10% fetal bovine serum (FBS) and 1% penicillin/streptomycin. Cells were passaged using 0.25% trypsin‐0.02% EDTA.

RNF31 siRNA sequences were synthesized by GENEPHARMA (Table S1). All Flag‐tagged RNF31 and His‐tagged TFEB truncation mutants were purchased from Haichuang Keye. Site‐directed mutagenesis was performed to generate Flag‐RNF31, His‐TFEB 2ST (S281A/T276A), TFEB S281D, T276D, and Flag‐RNF31 K908A plasmids; all constructs were verified by Sanger sequencing.

Details of primary antibodies are listed in Table S2. Secondary antibodies and magnetic beads were obtained from ThermoFisher and KATI LIFE. The inhibitors utilized in this study were as follows: LPS (Solarbio, L8880), MG132 (MedChemExpress, HY‐13259), Cycloheximide (CHX; MedChemExpress, HY‐12320), Alkaline phosphatase (ALP, 9001‐78‐9), and CM‐C1 (AbMole, M9451).

### 2.2 Animals and Colitis Model

#### 2.2.1 Generation of RNF31 Knockout Mice

Intestinal‐epithelial‐specific RNF31 knockout mice (Villin‐Cre/RNF31^fl/fl^, RNF31‐KO) and their littermate controls (RNF31^fl/fl^, CT) were generated on a C57BL/6 background (Liu, Zhang, et al. 2025). Briefly, RNF31 floxed mice were generated using CRISPR/Cas9 technology with loxP sites inserted flanking exons 4–5 of the RNF31 gene (genomic region: chr12: 27890000–27,910,000, GRCm38). These mice were then crossed with Villin‐Cre transgenic mice (expressing Cre recombinase specifically in intestinal epithelial cells) to obtain intestinal‐epithelial‐specific knockout mice. All experimental mice were 6–8 weeks old males, obtained from the Laboratory Animal Center of Nanchang University, and raised under specific pathogen‐free (SPF) conditions with a 12‐h light/dark cycle and free access to food and water.

Genotyping was performed by PCR on tail genomic DNA. The RNF31 floxed allele was detected using primers: forward 5′‐CCATGGAAGCCAGAACCGAT‐3′ (P1) and reverse 5′‐GCCAGATCCCTTAGCACTGG‐3′ (P2). The PCR products were 266 bp for the wild‐type allele and 386 bp for the floxed allele. The Villin‐Cre transgene was detected using a three‐primer system: forward 5′‐TTCATGATAGACAGATGAACACAGAT‐3′ (P4, wild‐type specific), common reverse 5′‐GCTTTCAAGTTTCATCCATGTGTG‐3′ (P5), and mutant‐specific reverse 5′‐GTCTTTGGGTAAAGCCAAGC‐3′ (P6), yielding a ~119 bp product for the Cre transgene.

#### 2.2.2 Animal Model and Treatment

Male C57BL/6 mice (6‐weeks‐old) were acclimatized for 1 week under standard conditions. Colitis was induced by three cycles of 2% dextran sulfate sodium (DSS; molecular weight 36–50 kDa; MP Biomedicals) in drinking water, each followed by a recovery period with sterile water, over a total of 10 weeks. The selection of CM‐C1 doses (10 and 25 mg/kg) was based on the following rationale: (i) conversion from the effective in vitro concentration using body surface area normalization; (ii) the safe and effective dose range (10–100 mg/kg) reported for CM‐C1 and its structural analogs in comparable models of inflammation (He et al. 2023, 2025; Cai, Jiang, et al. 2024); and (iii) a pilot tolerability study, which indicated that 10 and 25 mg/kg were well‐tolerated and showed preliminary anti‐inflammatory efficacy, whereas higher doses (40 and 100 mg/kg) induced adverse effects.

For intervention, mice received CM‐C1 at doses of 10 or 25 mg/kg, or an equal volume of vehicle, by oral gavage every other day (Hussain et al. 2022). Body weight, stool consistency, bleeding, and activity were monitored weekly for clinical disease scoring. At the endpoint, mice were euthanized, and intestinal tissues were collected for analysis (Zhang, Zhang, et al. 2024; Wang, Zhou, et al. 2024). All animal procedures were conducted in accordance with protocols approved by the Institutional Animal Care and Use Committee (IACUC) of The First Affiliated Hospital, Jiangxi Medical College, Nanchang University (Approval No. CDYFY‐IACUC‐202503QR002).

### 2.3 CCK8 Assay

Cells were seeded at 1 × 10^4^ cells/well and treated with LPS or CM‐C1. After 6–48 h, 10 μL of CCK‐8 reagent was added, and absorbance at 450 nm was measured. IC₅₀ values were calculated using GraphPad Prism. A preliminary dose–response CCK‐8 assay (Figure S1) indicated that CM‐C1 at 2 μg/mL effectively influenced cellular responses while exhibiting minimal cytotoxicity over 24 h. Based on these results, this concentration was selected for all subsequent in vitro mechanistic experiments.

### 2.4 Immunohistochemical (IHC) Staining

Tissue sections were dehydrated, subjected to citrate‐based antigen retrieval, and blocked with 3% goat serum for 1 h at room temperature. Sections were incubated with primary antibody at 4°C overnight, followed by DAB development, hematoxylin counterstaining, and ethanol dehydration. Stained sections were imaged using ImageScope software and evaluated independently by two pathologists in a blinded manner (Assa et al. 2025). Staining scores were calculated as the product of the positive cell proportion score (1: < 25%; 2: 25%–50%; 3: 50%–75%; 4: > 75%) and intensity score (0: negative; 1: light brown; 2: medium brown; 3: dark brown). Samples were classified as high or low expression based on a median total score threshold of 4.

### 2.5 Immunofluorescence (IF) Staining

Cells grown on coverslips treated as indicated (e.g., with 2.5 μg/mL LPS and/or 2.0 μg/mL CM‐C1 for 24 h), then fixed with 4% paraformaldehyde, while tissues were fixed, dehydrated, and embedded. Samples were permeabilized with Triton X‐100, blocked with serum, and incubated with primary antibodies at 4°C overnight. After washing, fluorescent‐labeled secondary antibodies were applied, and nuclei were stained with DAPI. Images were acquired using a fluorescence microscope.

### 2.6 Immunoprecipitation (IP) and Western Blot (WB) Assays

NCM460 cells were treated with 2.0 μg/mL CM‐C1, alone or in combination with 2.5 μg/mL LPS, for 24 h as indicated. Subsequently, cells were lysed in NETN buffer and centrifuged at 12,000 × g for 15 min. Supernatants were incubated with Anti‐Flag Nanobody Magarose beads overnight at 4°C. Beads were washed, and bound proteins were eluted with SDS loading buffer for immunoblotting. Proteins were separated by SDS‐PAGE, transferred to NC membranes, blocked with 5% BSA, and probed with primary antibodies overnight at 4°C. Protein bands were visualized and quantified using Image Lab software (Bio‐Rad).

### 2.7 Ubiquitination Assays

For denaturing IP, cells were lysed in Triton X‐100 buffer, diluted, and centrifuged. Lysates were immunoprecipitated and immunoblotted with anti‐ubiquitin antibody. For in vitro ubiquitination, purified His‐TFEB and Flag‐RNF31 from HEK293T cells were incubated with FastAP alkaline phosphatase (Thermo Scientific), ubiquitin (Hitrobio), and reaction buffer overnight at 4°C. Products were analyzed by SDS‐PAGE and immunoblotting (Yan et al. 2024).

### 2.8 RNA Isolation and Quantitative RT‐PCR

Total RNA was extracted using Vazyme RNeasy columns and reverse‐transcribed into cDNA. qPCR was performed using SYBR Green reagent on an ABI 7500 Fast system. Gene expression was normalized to GAPDH and calculated via the 2^−ΔΔCT^ method. Primer sequences are listed in Table S3.

### 2.9 Transmission Electron Microscopy (TEM)

Tissue pellets were fixed in TEM fixative, pre‐embedded in agarose, and post‐fixed with 1% OsO_4_. Samples were dehydrated through an ethanol‐acetone series, infiltrated with EMBed 812 resin, and polymerized at 60°C. Ultrathin sections (60–80 nm) were stained with uranyl acetate and lead citrate, and imaged under a TEM.

### 2.10 Membrane and Nuclear Protein Extraction

Membrane and nuclear proteins were extracted using commercial kits (Good Laboratory Practice Bioscience) according to the manufacturer's instructions. Briefly, cells or tissues were lysed, and fractionation was performed by differential centrifugation. Extracted proteins were stored at −80°C.

### 2.11 Elisa

Supernatants were collected from NCM460 cells treated with LPS (2.5 μg/mL) and/or CM‐C1 (2.0 μg/mL) for 24 h. Levels of IL‐6, IL‐1β, and TNF‐α were measured using commercial ELISA kits (Boster Biological Technology) according to the manufacturer's protocol. Absorbance was read at 450 nm, and cytokine concentrations were determined from standard curves. All extractions were performed using commercial kits (Boster Biological Technology, Wuhan, China) according to the manufacturers' instructions (Table S4).

### 2.12 GFP‐mRFP‐LC3 Autophagic Flux Assay and Bafilomycin A1 Treatment

NCM460 cells were transfected with the GFP‐mRFP‐LC3 tandem reporter plasmid (Hanbio, China) using Lipofectamine 3000 (Thermo Fisher). After 24 h, cells were treated with LPS (2.5 μg/mL), CM‐C1 (2.0 μg/mL), and/or bafilomycin A1 (Baf A1, 100 nM, MedChemExpress, HY‐100558) for an additional 24 h. Cells were then fixed with 4% paraformaldehyde, and images were acquired using a confocal microscope (Zeiss LSM880). GFP (green) and mRFP (red) puncta were counted using ImageJ software.

### 2.13 Screening for RNF31‐Interacting Proteins and Clinical Relevance Analysis

To identify potential interacting partners of the E3 ubiquitin ligase RNF31 associated with IBD pathogenesis, we performed an integrative bioinformatics screening using multiple databases and a stepwise filtering strategy, as detailed below.

#### 2.13.1 Database Sources, Versions, and Parameters

UbiBrowser 2.0 (confidence score ≥ 0.5, species: *Homo sapiens* ), GeneMANIA (2018 release, default settings), GeneCard (V5.21), BioGRID (4.4.246).

#### 2.13.2 Screening Strategy and Parameters

The screening employed a multi‐step approach. First, candidate RNF31‐interacting proteins were predicted using the following tools:

- UbiBrowser 2.0: using RNF31 (Entrez Gene ID: 55072) as the query E3 ligase, retaining the top 100 predicted substrates with a confidence score ≥ 0.5;
- GeneMANIA: selecting the top 50 functionally related genes based on physical interaction and co‐expression networks;
- GeneCard: retrieving interactors annotated as “interacts with RNF31” or predicted based on domain information;
- BioGRID: retrieving all experimentally validated physical and genetic interactors of RNF31 in *H. sapiens* supported by at least one peer‐reviewed publication.

Subsequently, candidate proteins from the four databases were combined, and only those identified by at least three of the four databases were retained as high‐priority candidates for further validation. Ranked by the frequency of intersection, TFEB (Entrez Gene ID: 79442) and CYLD (Entrez Gene ID: 1540) were the top two. TFEB, a master regulator of autophagy and lysosomal biogenesis, directly links RNF31 to autophagic dysfunction and has been implicated in IBD pathogenesis; it was therefore prioritized. CYLD, a deubiquitinase that regulates NF‐κB and inflammatory signaling pathways, was selected as a positive control for ubiquitin‐related pathways.

#### 2.13.3 GSEA of TFEB‐Associated Inflammatory Pathways

To evaluate the clinical relevance of TFEB in IBD, GSEA was performed on the GSE75214 dataset (GPL6244 platform, 172 IBD patients vs. 22 healthy controls (NC)). Differentially expressed genes were defined as FDR < 0.05 and |log_2_FC| > 1. Enrichment analysis used MSigDB C5 gene sets (focusing on GOBP) with GSEA software v4.3.2 (1000 permutations, gene set size 15–500). Significance was set at FDR q < 0.05 and |NES| > 1.5.

#### 2.13.4 Inclusion/Exclusion Criteria and Candidate Ranking Logic for Validation

Candidate genes were included if identified by ≥ 3 databases and annotated with relevance to intestinal inflammation or ubiquitin‐proteasome pathway. Exclusion criteria: uncharacterized proteins or single‐database hits with confidence < 0.5. The composite score ranked TFEB as the top candidate, with CYLD as a positive control. The complete results of the bioinformatics screening, including the full list of candidate RNF31‐interacting proteins with their database sources, confidence scores, functional annotations, and ranking calculations, are provided in Table S5.

### 2.14 Protein Structure Prediction and Interaction Interface Analysis

To predict the three‐dimensional structure of the RNF31–TFEB complex and identify key interacting residues, the amino acid sequences of human RNF31 (UniProt ID: Q9Y4X5) and TFEB (UniProt ID: P19484) were retrieved from the UniProt database (https://www.uniprot.org/). The sequences were submitted to the AlphaFold3 online server (version 1.0, https://deepmind.google/technologies/alphafold/alphafold‐server/) for protein–protein interaction structure prediction using default parameters (five seeds, full database). Five interaction models were generated, and the model with the optimal ranking score (Model 0) was selected for interface analysis. Model confidence was assessed by the predicted Local Distance Difference Test (pLDDT) score; the selected model showed high confidence with an average pLDDT > 90 in the interface region. The predicted interaction interface was visualized using PyMOL 2.6 (open‐source version), and hydrogen bonds as well as key residues (TFEB S281/T276 and RNF31 K908) involved in specific interactions were identified and annotated.

### 2.15 Gut Microbiome Analysis

Fecal samples were collected from mice in each group (*n* = 5 per group) at the endpoint of the experiment. Microbial genomic DNA was extracted using a commercial stool DNA kit (e.g., MagPure Stool DNA Kit, Magen, China) according to the manufacturer's instructions. The V3–V4 hypervariable regions of the bacterial 16S rRNA gene were PCR‐amplified using primers 338F (5′‐ACTCCTACGGGAGGCAGCA‐3′) and 806R (5′‐GGACTACHVGGGTWTCTAAT‐3′). Amplicons were separated by 2% agarose gel electrophoresis, excised, and purified using Vazyme VAHTSTM DNA Clean Beads. Sequencing libraries were constructed with the TruSeq Nano DNA LT Library Prep Kit (Illumina, USA) and sequenced on an Illumina platform by Wuhan Servicebio Technology Co. Ltd. (China). Paired‐end 250 bp reads were generated.

Raw sequencing data were quality‐filtered using Trimmomatic (v0.39): reads with an average quality score < Q20 over a 50 bp sliding window were truncated, and reads shorter than 200 bp were discarded. Chimeric sequences were removed using VSEARCH (v2.13.4). After quality control, an average of 52,341 ± 3204 high‐quality reads per sample were obtained. The high‐quality reads were clustered into operational taxonomic units (OTUs) at 97% sequence identity using UPARSE (v7.1). Taxonomy was assigned using the SILVA database (v138) with a confidence threshold of 0.7.

Bioinformatics analysis was performed using QIIME2 (version 2023.9) and R (version 4.3.1). To assess inter‐group differences in microbial community structure (β‐diversity), weighted and unweighted UniFrac distances were calculated, followed by visualization of the resulting distance matrices using principal coordinate analysis (PCoA). To evaluate within‐sample (alpha) diversity, we employed the Chao1 index, the Shannon index, and the ACE index. Linear discriminant analysis Effect Size (LEfSe) was used to identify differentially abundant taxa (LDA score > 2.0, *p* < 0.05).

### 2.16 Statistical Analysis

The normality of continuous data was assessed using the Shapiro–Wilk test or by visual inspection of Q–Q plots. Normally distributed data (*p* ≥ 0.05) were analyzed with parametric tests: an unpaired two‐tailed Student's *t*‐test for comparisons between two groups, or one‐way ANOVA followed by Tukey's post hoc test for multiple comparisons. Non‐normally distributed data were analyzed with corresponding non‐parametric tests: the Mann–Whitney *U* test for two groups, or the Kruskal–Wallis test followed by Dunn's post hoc test for multiple groups. Data are presented as box plots or as mean ± SEM, as appropriate. All statistical analyses were performed using GraphPad Prism software (version 10). A *p*‐value < 0.05 was considered statistically significant.

## 3 Results

### 3.1 TFEB Is Identified as a Clinically Relevant RNF31‐Interacting Protein

**FIGURE 1:**
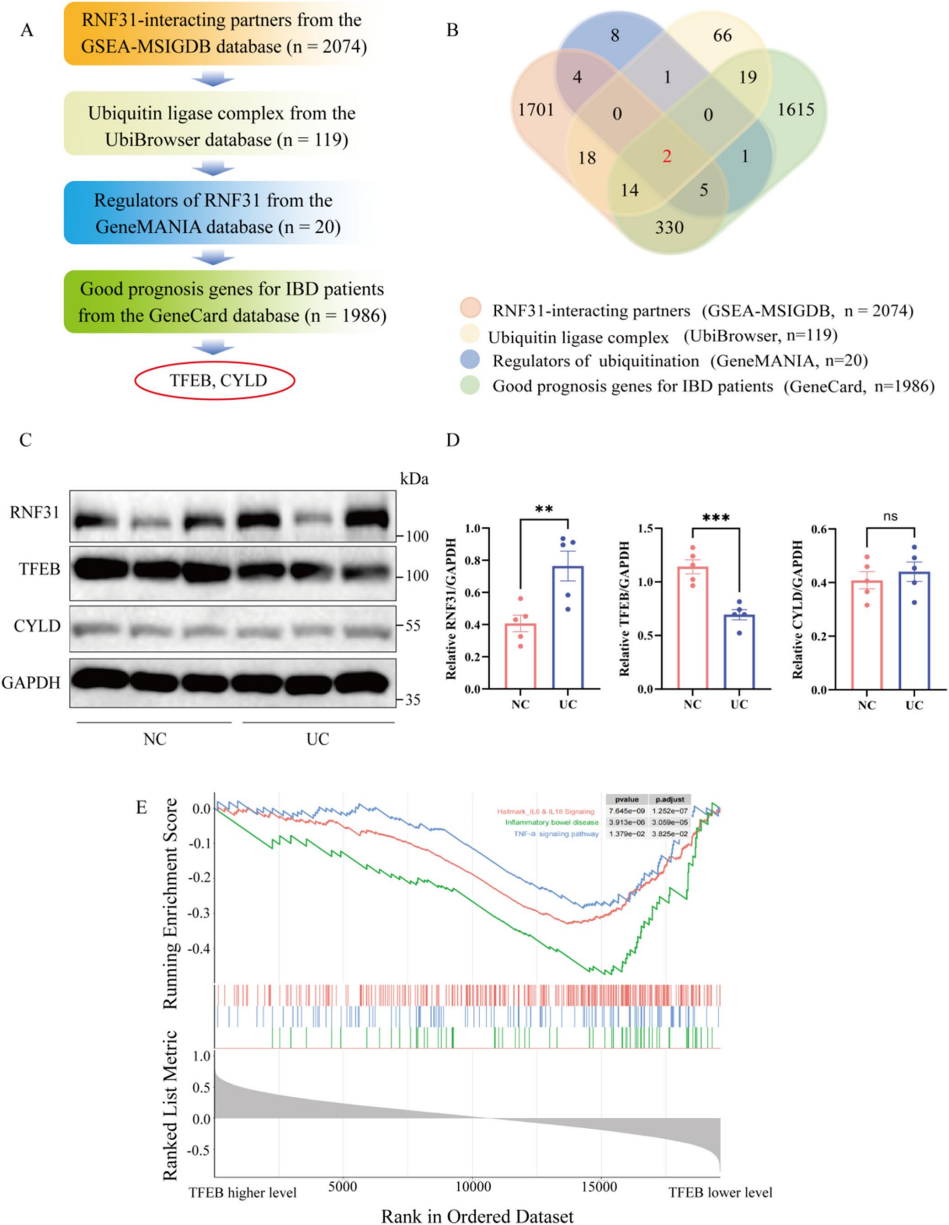
*Identification of TFEB as an RNF31‐interacting protein and its role in LPS‐induced signaling*. (A) Schematic of the screening strategy to identify TFEB as a putative RNF31 substrate. (B) Venn diagram showing the overlap of candidate proteins from the UbiBrowser, GeneMANIA, GeneCard, and GSEA‐MSigDB databases. (C, D) Representative protein expression (C) and quantitative analysis (D) of RNF31, CYLD, and TFEB in colonic tissues from normal control (NC) and UC patients (*n* = 5 per group). (E) Gene set enrichment analysis (GSEA) plot showing enrichment of inflammatory bowel disease (IBD) and inflammatory pathways in samples with low TFEB expression. Data are presented as mean ± SEM. Statistical significance was determined by Student's *t*‐test (**p < 0.01).

Based on integrated bioinformatic screening, TFEB and CYLD were selected as top candidate interactors of RNF31 for experimental validation (Figure 1A,B). Comparative analysis of colonic tissues from healthy controls (NC) and patients with UC, which exhibited elevated RNF31 levels, demonstrated a marked decrease in TFEB protein levels in UC tissues, whereas CYLD expression remained unchanged (Figure 1C,D) Consistent with this, GSEA database revealed that gene sets related to IBD and inflammatory pathways were significantly enriched among proteins differentially expressed in correlation with low TFEB levels (Figure 1E), suggesting a potential role for TFEB in these processes. This inverse relationship hints at a functional interplay between RNF31 and TFEB in the intestinal context.

### 3.2 LPS Induces TFEB Phosphorylation and Inhibits Its Nuclear Translocation

**FIGURE 2:**
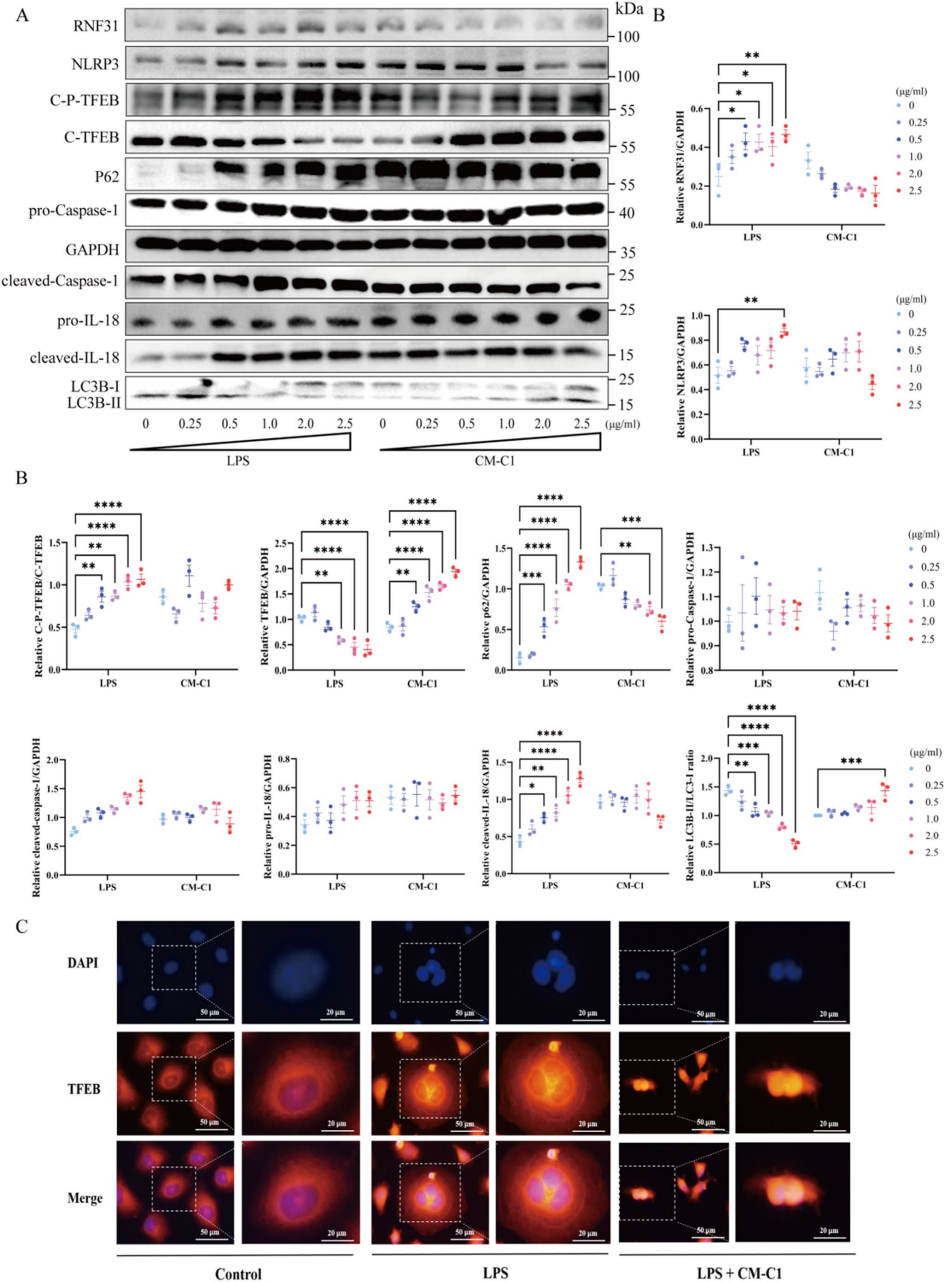
*Effects of LPS and CM‐C1 on protein expression and TFEB localization in vitro*. (A) Western blot analysis of relative protein expression in NCM460 cells treated for 24 h with LPS or CM‐C1 (B) Quantification of protein levels from (A). (C) Representative immunofluorescence images showing TFEB (red) and nuclei (DAPI, blue). Data are presented as mean ± SEM from three independent biological experiments (*n* = 3). Statistical significance was determined by one‐way ANOVA (*p < 0.05, **p < 0.01, ***p < 0.001, ****p < 0.0001).

To model the inflammatory milieu of IBD, we treated NCM460 with bacterial LPS. Dose–response experiments determined that 2.5 μg/mL LPS for 24 h and 2.0 μg/mL CM‐C1 were optimal for subsequent studies (Figure S1). At this concentration, LPS significantly enhanced the phosphorylation of TFEB at Ser211, concurrently reducing its accumulation in the nucleus (Figure 2A,B and Figure S1). This mislocalization was associated with a suppression of autophagic flux, evidenced by a decreased LC3B‐II/I ratio and an accumulation of SQSTM1/p62 (Figure 2A,B). Furthermore, LPS treatment potently upregulated key inflammatory mediators, including cleaved‐IL‐18 and cleaved‐Caspase‐1 (Figure 2A,B). IF analysis visually confirmed these findings, showing a pronounced diminution of TFEB fluorescence within the nucleus and its concomitant accumulation in the cytoplasm upon LPS challenge (Figure 2C).

### 3.3 RNF31 Interacts With and Promotes the Ubiquitination of TFEB

**FIGURE 3:**
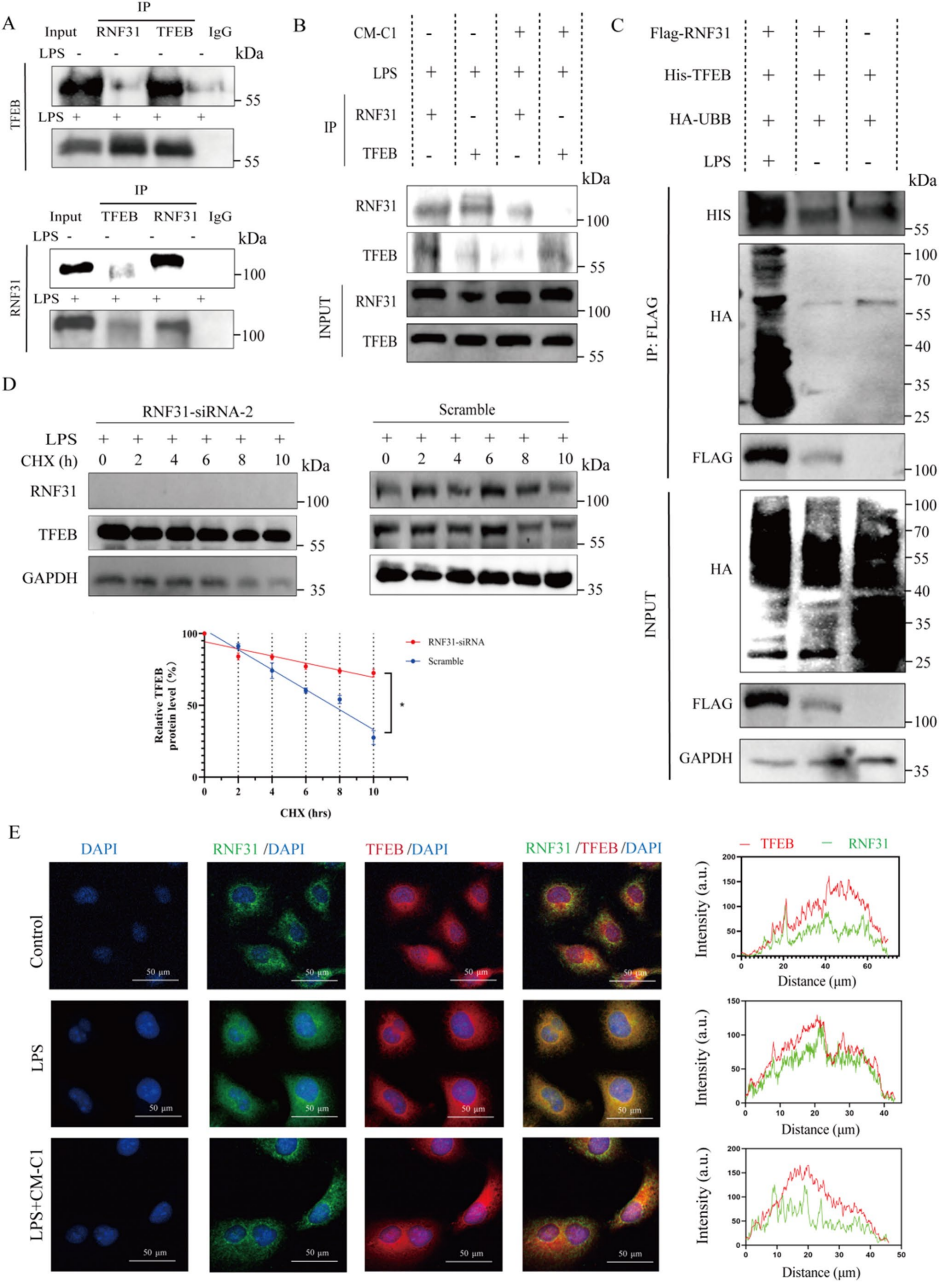
*RNF31 interacts with and ubiquitinates TFEB under LPS stimulation*. (A) Reciprocal co‐immunoprecipitation (Co‐IP) of endogenous TFEB and RNF31 in NCM460 cells. IgG served as a negative control. (B) Co‐IP analysis of TFEB and RNF31 in cells treated with LPS and/or CM‐C1. (C) Ubiquitination assay in HEK293T cells expressing Flag‐RNF31, His‐TFEB, and HA‐Ub, treated with MG132 and LPS. (D) (Top) Cycloheximide (CHX) chase assay in NCM460 cells transfected with scramble or RNF31‐siRNA‐2. (Bottom) Quantification of TFEB protein half‐life. The half‐life was calculated by fitting the data to a one‐phase exponential decay model, and the difference between conditions was compared using an extra sum‐of‐squares F‐test (*p < 0.05). (E) Immunofluorescence images showing co‐localization of RNF31 (red) and TFEB (green). Fluorescence intensity profiles along the indicated line are shown.

We next investigated the functional consequence of the RNF31‐TFEB interaction. Co‐IP assays in NCM460 cells confirmed a robust interaction between endogenous RNF31 and TFEB, which was markedly enhanced under LPS stimulation (Figure 3A,B). Given that LPS did not significantly alter TFEB mRNA levels (Figure S1), we hypothesized post‐translational regulation. Indeed, knockdown of RNF31 by siRNA led to a marked increase in TFEB protein levels (Figure S1), whereas siRNA‐mediated knockdown of TFEB did not affect RNF31 levels (Figure S1), indicating a negative regulatory relationship placing RNF31 upstream of TFEB. Crucially, upon co‐expression of Flag‐RNF31, His‐TFEB, and HA‐Ubiquitin in HEK293T cells, LPS treatment dramatically enhanced the ubiquitination of TFEB (Figure 3C). A CHX chase assay provided further mechanistic insight, revealing that RNF31 depletion significantly prolonged the half‐life of the TFEB protein (Figure 3D), confirming that RNF31 regulates TFEB stability via the ubiquitin‐proteasome pathway. Additionally, LPS promoted the co‐localization of TFEB and RNF31 in the cytoplasm; however, this LPS‐induced co‐localization was effectively blocked by CM‐C1 treatment (Figures 2C and 3E).

### 3.4 Mapping the Critical Interaction and Ubiquitination Sites Between RNF31 and TFEB

**FIGURE 4:**
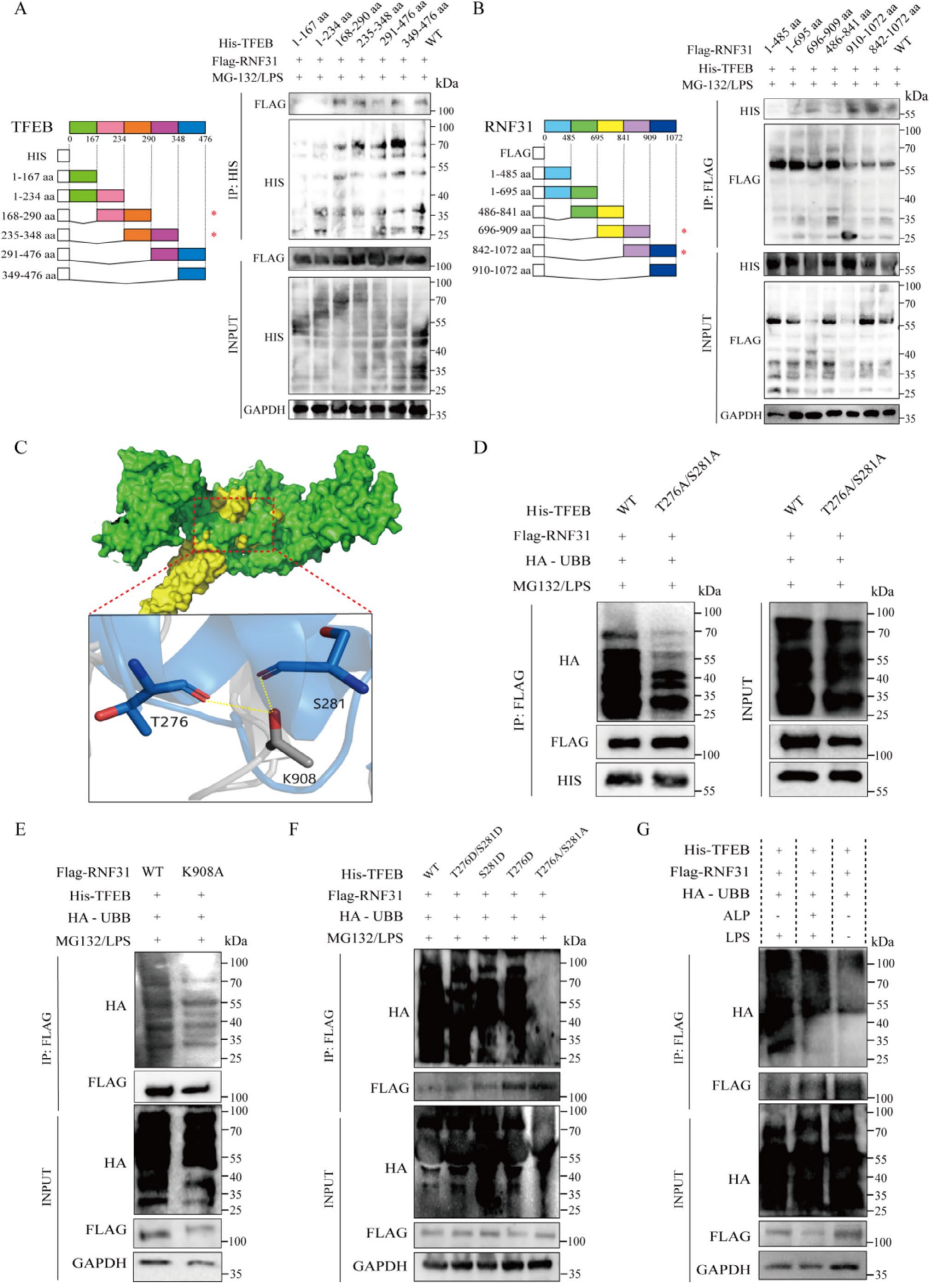
*Ping the RNF31‐TFEB interaction and ubiquitination sites*. (A) Schematics of Flag‐tagged RNF31 and His‐tagged TFEB truncation mutants () Co‐IP assays of full‐length RNF31 with TFEB truncation mutants (left) and full‐length TFEB with RNF31 truncation mutants (right) (B). (C) Molecular docking model of the RNF31 (green)–TFEB (yellow) complex. Key interacting residues and hydrogen bonds are indicated by dashed lines. (D, E) Ubiquitination assays in HEK293T cells co‐expressing HA‐Ub with wild‐type or mutant forms of TFEB (D) and RNF31 (E). (F) Analysis of ubiquitination levels of TFEB phospho‐mutants (S281A, T276A, S281D, T276D). (G) In vitro ubiquitination assay using dephosphorylated (alkaline phosphatase, ALP‐treated) or phosphorylated (LPS‐treated) TFEB.

To delineate the molecular details of this interaction, we performed a series of mapping experiments. Co‐IP assays with various truncation mutants identified the 235–290 amino acid region of TFEB as responsible for binding to RNF31 (Figure 4A), and conversely, the 842–909 amino acid region of RNF31 as the primary domain interacting with TFEB (Figure 4B). Bioinformatic analysis via BioGRID and GPS‐Uber predicted that TFEB residues T276 and S281 are adjacent to RNF31's K908 residue, potentially forming stabilizing hydrogen bonds (Figure 4C). Functional validation through ubiquitination assays showed that a TFEB double mutant (S281A/T276A, termed 2ST) almost completely abolished RNF31‐mediated ubiquitination (Figure 4D). Similarly, a single point mutation of the RNF31 interaction site (K908A) also effectively eliminated ubiquitination (Figure 4E). Employing phosphomimetic (S281D, T276D) and phosphodead (S281A, T276A) mutants, we demonstrated that mimicking phosphorylation enhanced TFEB ubiquitination, while mimicking dephosphorylation reduced it (Figure 4F). Finally, an in vitro ubiquitination assay confirmed that ALP‐mediated dephosphorylation of TFEB significantly attenuated its ubiquitination by RNF31 (Figure 4G), establishing that LPS‐induced phosphorylation is a prerequisite for efficient ubiquitination.

### 3.5 CM‐C1 Disrupts the RNF31‐TFEB Axis to Alleviate Inflammation

**FIGURE 5:**
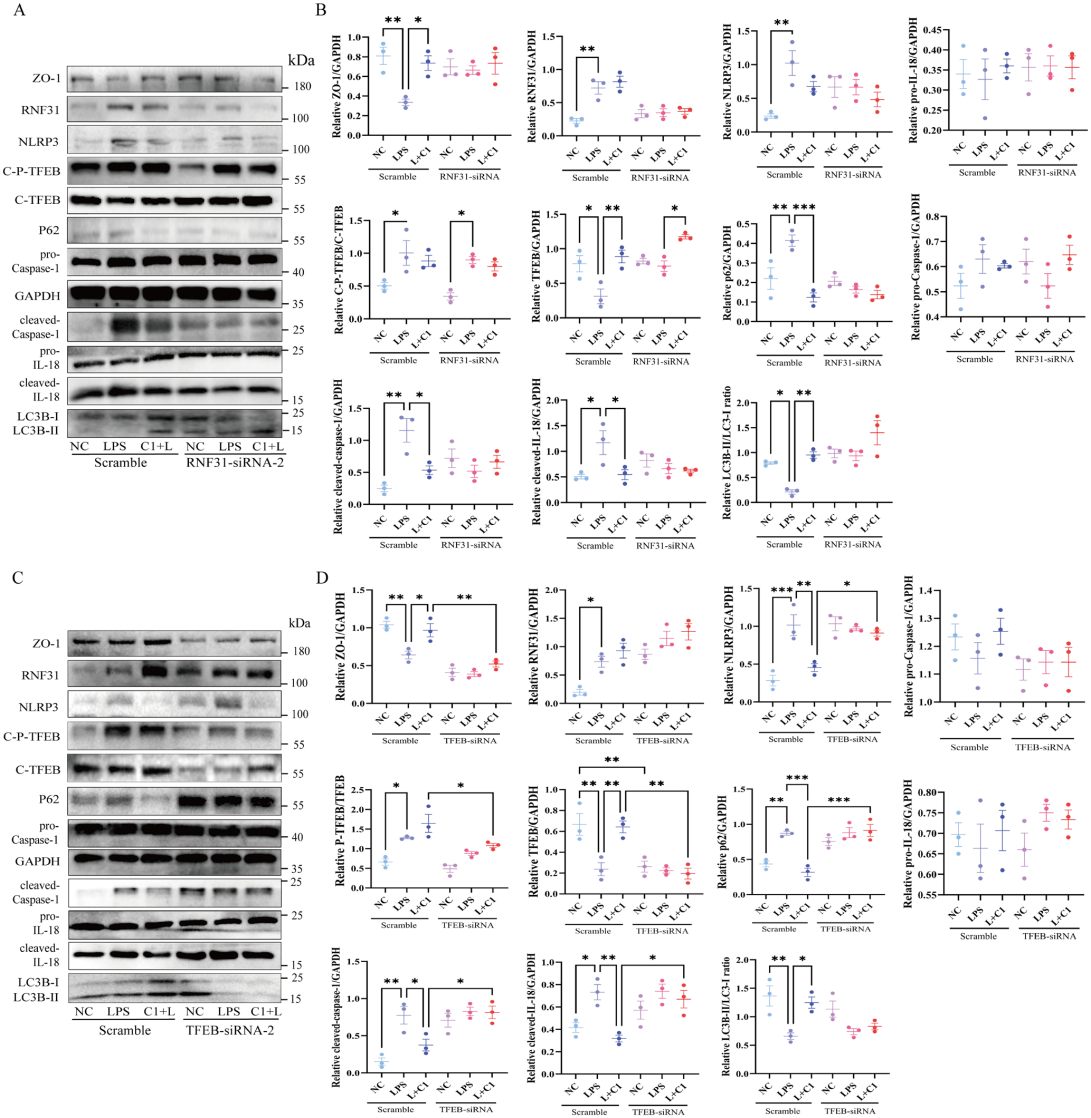
*Functional interrogation of the RNF31‐TFEB axis in LPS‐induced signaling*. NCM460 cells were treated as follows: NC (normal control); LPS (2.5 μg/mL, 24 h); C1 + L (co‐treatment with CM‐C1 (2.0 μg/mL) and LPS (2.5 μg/mL) for 24 h). (A) Western blot analysis of cells transfected with RNF31‐siRNA and treated with LPS and/or CM‐C1. (B) Quantification of relative protein levels from (A). (C) Western blot analysis of cells transfected with TFEB‐siRNA and treated with LPS and/or CM‐C1. (D) Quantification of relative protein levels from (C). Data are presented as mean ± SEM from three independent biological experiments (*n* = 3). Statistical significance was determined by one‐way ANOVA (*p < 0.05, **p < 0.01, ***p < 0.001).

Experimental results showed that CM‐C1 treatment effectively reversed the abnormal phenotypes induced by LPS: it significantly promoted TFEB nuclear accumulation without affecting TFEB phosphorylation status, restored cellular autophagic flux, and markedly down‐regulated the expression levels of cleaved‐IL‐18, cleaved‐Caspase‐1, and NLRP3 (Figure 5A,B); notably, CM‐C1 did not affect the LPS‐induced up‐regulation of RNF31 itself (Figure 5A,B). To further verify whether the core mechanism of CM‐C1 in inhibiting LPS‐induced inflammation depends on the specific regulation of TFEB, we constructed a stably transfected NCM460 cell line with TFEB knockdown (Figure S1). The results demonstrated that when the TFEB gene was inhibited, even with CM‐C1 treatment, its anti‐inflammatory effect was completely abolished, and the cells still maintained a pro‐inflammatory phenotype similar to that of the LPS group, specifically characterized by low autophagic activity and high expression of inflammatory factors; in addition, TFEB inhibition itself directly interfered with cellular autophagic function, triggered a pro‐inflammatory state, and impaired intestinal barrier integrity, as evidenced by the down‐regulated expression of the tight junction protein ZO‐1 (Figure 5C,D). Collectively, these data establish that LPS exerts its pro‐inflammatory effects by activating the RNF31/TFEB axis, while CM‐C1 counteracts this pathway by targeting TFEB.

Additionally, we employed a GFP‐mRFP‐LC3 tandem reporter. LPS treatment significantly reduced both GFP and mRFP puncta, indicating impaired autophagosome formation and autophagic flux. In contrast, CM‐C1 treatment markedly increased yellow (merged) puncta, reflecting enhanced autophagosome generation (Figure S2A,B). To distinguish between autophagosome synthesis and degradation, we co‐treated cells with Baf A1, a lysosomal inhibitor that blocks autophagic degradation. In CM‐C1‐treated cells, Baf A1 further augmented the accumulation of mRFP‐positive puncta (while GFP signal was quenched in acidic compartments), confirming that CM‐C1 indeed promotes autophagic flux rather than simply blocking autophagic degradation (Figure S2A,B).

To explore whether the suppressive effect of CM‐C1 on NLRP3 inflammasome activation involves autophagic restoration, we treated cells with Baf A1 in the presence of LPS plus CM‐C1. Baf A1 treatment reversed the CM‐C1‐induced reduction of NLRP3 protein levels, leading to a marked re‐accumulation of NLRP3 (Figure S2C,D). This observation is consistent with the idea that intact autophagic degradation contributes to the anti‐inflammatory action of CM‐C1, although it does not rule out other autophagy‐independent effects of Baf A1.

### 3.6 CM‐C1 Ameliorates DSS‐Induced Colitis in Mice by Enhancing Autophagy and Restoring Gut Barrier Function

**FIGURE 6:**
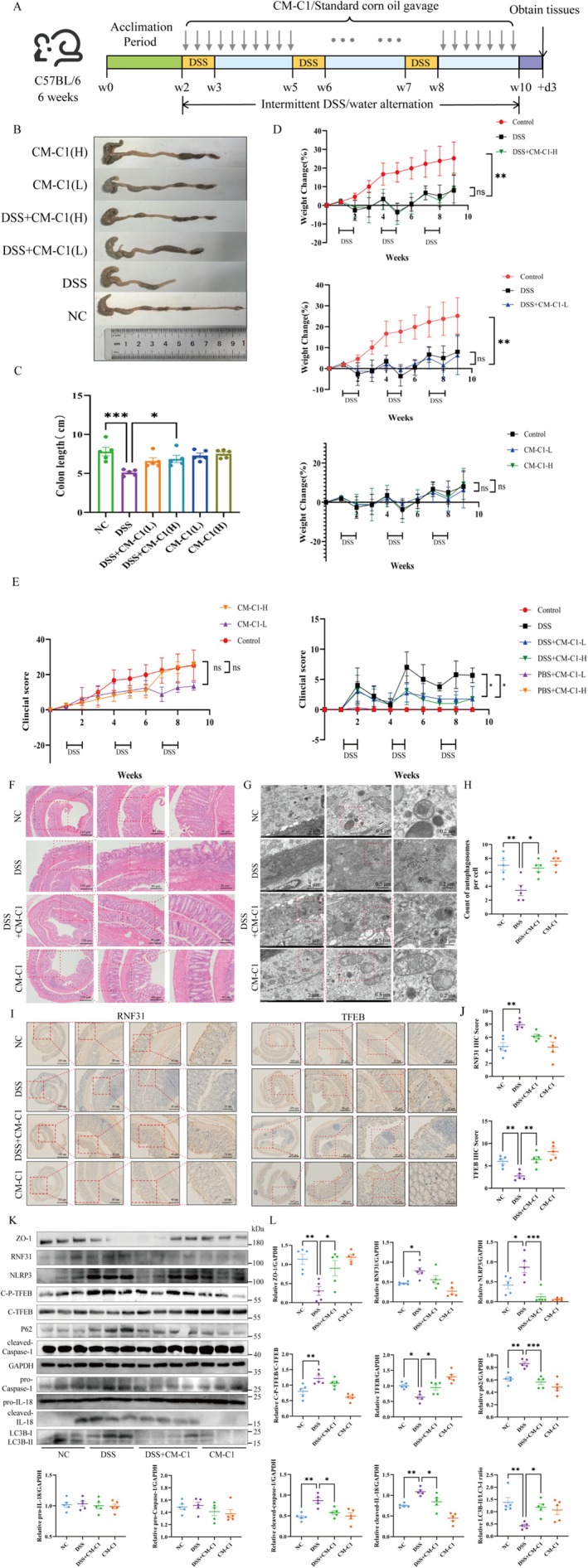
*CM‐C1 alleviates DSS‐induced colitis in mice*. (A) Experimental timeline of dextran sulfate sodium (DSS) and CM‐C1 administration. (B, C) Representative images of colons (B) and colon length measurements (C) from each group. (D, E) Body weight change (D) and clinical disease activity index (DAI) scores (E) during the experiment. (F) Representative H&E‐stained colon sections. (G) Transmission electron micrographs of intestinal epithelial cells. (H) Quantification of autophagosomes per cell. (I, J) Representative IHC staining (I) and quantification of RNF31 and TFEB (J) in colon tissues. (K, L) Western blot analysis (K) and quantification of relative protein levels (L) in colon tissues (*n* = 5 per group). NC, normal control; CM‐C1 (L), low dose (10 mg/kg); CM‐C1 (H), high‐dose (25 mg/kg). Data are presented as mean ± SEM (*n* = 5 mice per group); each data point in panels C, D, E, H, J, and L represents a single mouse. Statistical significance was determined by one‐way ANOVA (*p < 0.05, **p < 0.01, ***p < 0.001).

In a murine model of UC induced by cyclic administration of DSS, CM‐C1 treatment demonstrated significant protective effects (Figure 6A). While it did not fully prevent DSS‐induced colon shortening (Figure 6B,C) or weight loss (Figure 6D), it significantly improved the disease activity index, particularly ameliorating bloody stools, diarrhea, and lethargy (Figure 6E). It revealed that CM‐C1 preserved intestinal mucosal architecture, reduced ulceration, and diminished inflammatory cell infiltration compared to the DSS‐only group (Figure 6F). TEM results confirmed a functional outcome, showing a significant increase in the number of autophagosomes in the colonic epithelium of the CM‐C1 + DSS group (Figure 6G,H). IHC analysis of colonic tissues showed that CM‐C1 treatment elevated TFEB protein levels and promoted the expression of the tight junction proteins ZO‐1, thereby restoring gut barrier integrity (Figure 6I–L, Figure S3A–D). CM‐C1 also significantly enhanced autophagic activity, as evidenced by an increased LC3B‐II/I ratio and decreased p62 expression (Figure 6K,L); meanwhile, it effectively regulated DSS‐induced inflammatory responses, which is reflected by reduced cleaved‐IL‐18 levels and a marked downregulation of the pro‐inflammatory cytokines IL‐6, IL‐1β, and tumor necrosis factor‐α (TNF‐α) in plasma (Figure S3E).

To further substantiate the essential role of RNF31 in regulating autophagy and gut barrier function, we utilized RNF31‐KO mice. Successful generation of intestinal‐epithelial‐specific RNF31 knockout was confirmed by Western blotting and qPCR (Figure S4A–C). Moreover, IHC staining revealed that DSS treatment decreased TFEB expression in CT mice but increased it in RNF31‐KO mice (Figure S4D–G). DSS treatment in CT control mice led to markedly reduced LC3B fluorescence intensity, indicating suppressed autophagy. In contrast, RNF31‐KO mice subjected to DSS exhibited significantly higher LC3B intensity, comparable to that in untreated CT mice, demonstrating that RNF31 deficiency effectively blocks DSS‐induced autophagic suppression (Figure S4H,I). Consistently, the expression of the tight junction protein ZO‐1 was also diminished in DSS‐treated CT mice, whereas RNF31‐KO mice maintained robust ZO‐1 levels under DSS challenge (Figure S4J,K).

### 3.7 CM‐C1 Modulates the Gut Microbiota Composition in DSS‐Treated Mice

**FIGURE 7:**
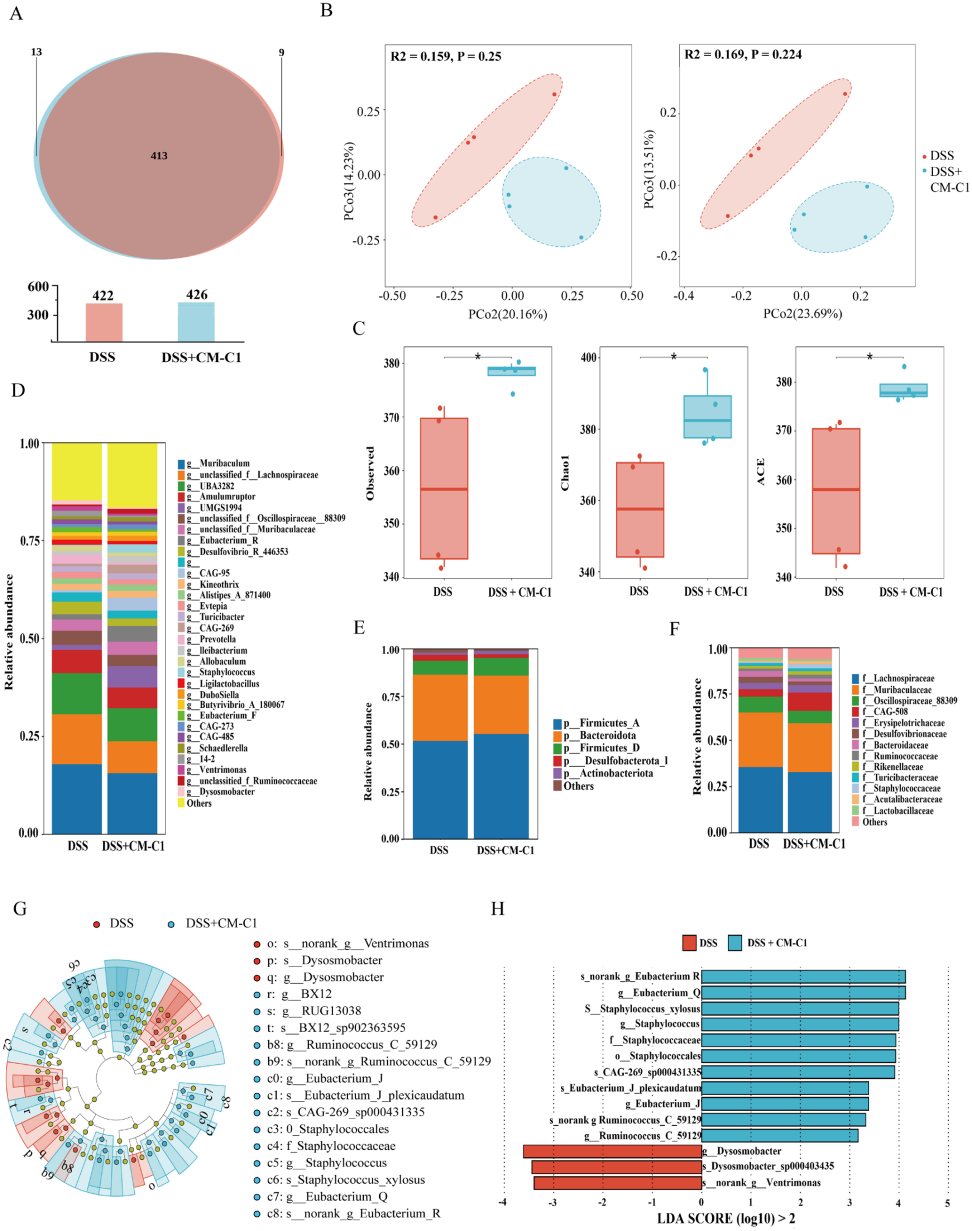
*CM‐C1 modulates the gut microbiota in DSS‐induced colitis*. (A) Venn diagram of operational taxonomic units (OTUs) shared between the DSS and CM‐C1 + DSS groups. (B) Principal coordinates analysis (PCoA) and non‐metric multidimensional scaling (NMDS) plots of gut microbiota composition. (C) Alpha diversity indices (observed species, Chao1, Ace). (D–F) Relative microbial abundance at the phylum (D), genus (E), and family (F) levels. (G, H) Linear discriminant analysis effect size (LEfSe) identifying differentially abundant bacterial taxa between groups (LDA score > 2, *p* < 0.05). Fecal samples were collected from *n* = 4 mice per group for sequencing; sequencing depth was normalized to ~50,000 reads per sample. In panel C (alpha diversity indices), each dot represents an individual mouse. Data are presented as mean ± SEM. Statistical significance was determined by Student's *t*‐test. (*p < 0.05).

**FIGURE 8:**
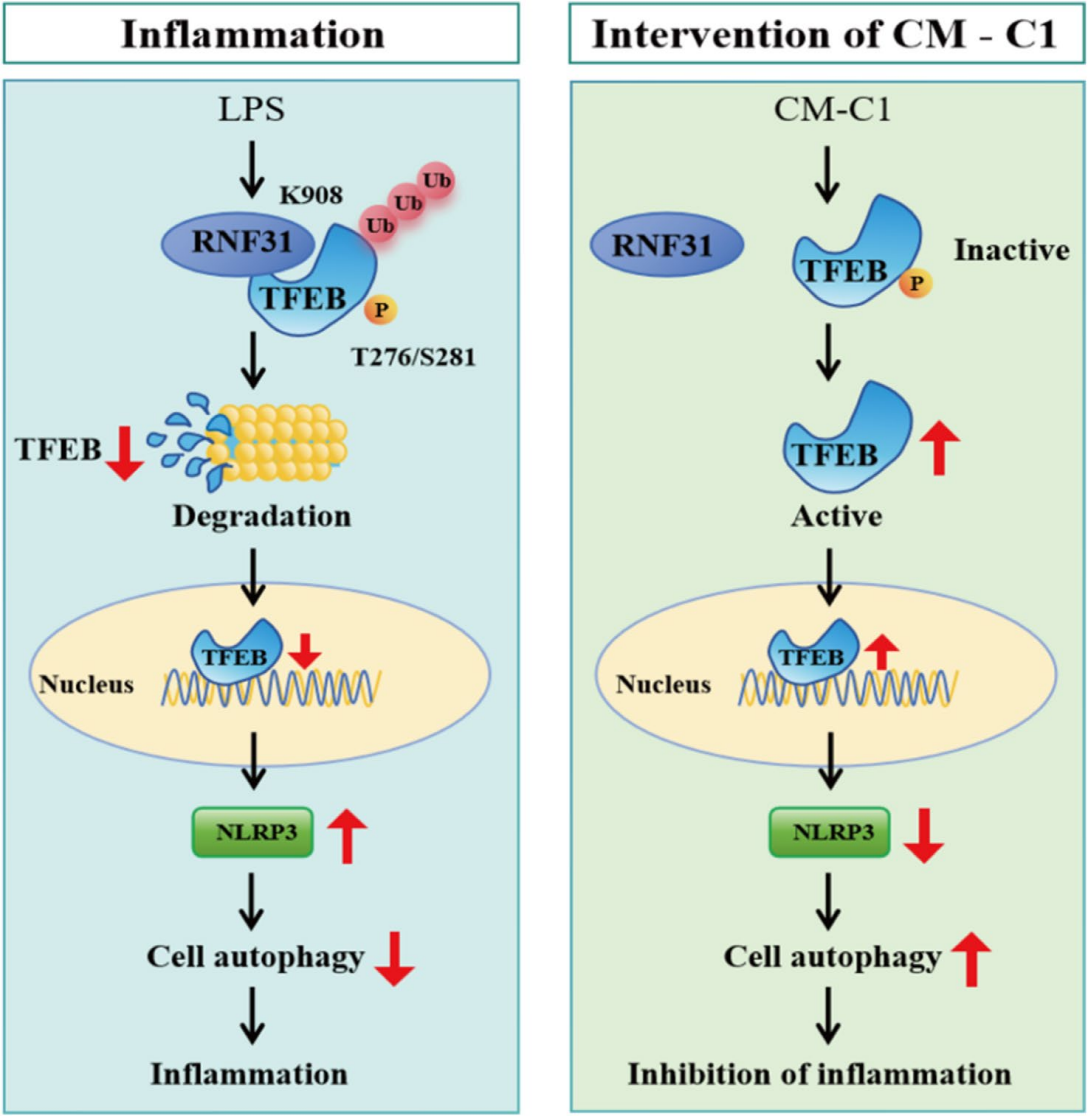
*Proposed model: CM‐C1 disrupts the RNF31–TFEB–NLRP3 axis to alleviate intestinal inflammation*. The model illustrates that LPS‐induced phosphorylation of TFEB promotes its binding to RNF31 (via TFEB‐S281/T276 and RNF31‐K908), leading to TFEB ubiquitination and degradation. This process suppresses autophagy while promoting NLRP3 inflammasome activation and IL‐18 secretion. CM‐C1 disrupts this axis by promoting TFEB nuclear translocation, thereby restoring autophagy and suppressing inflammation.

Given the link between host inflammation and gut microbiota, we analyzed fecal samples via 16S rRNA sequencing. Beta‐diversity analysis, assessed using PCoA and non‐metric multidimensional scaling (NMDS), revealed a clear separation between the microbial community structures of the CM‐C1 + DSS and DSS‐only groups (Figure 7B,C), indicating that CM‐C1 induces a significant shift in the gut microbiota. At the phylum level, CM‐C1 intervention increased the relative abundance of *Firmicutes* and decreased that of *Bacteroidota* (Figure 7D). Shifts were also evident at the genus and family levels (Figure 7E,F). Linear Discriminant Analysis Effect Size (LEfSe) identified specific bacterial taxa associated with each group. The CM‐C1 + DSS group was enriched for beneficial genera such as *Eubacterium*, *Staphylococcus*, and *Ruminococcus*, while the DSS group was enriched for *Dysomobacter* and *Ventrimonas* (LDA score > 3.0, *p* < 0.05; Figure 7G,H). These findings suggest that the therapeutic effect of CM‐C1 is partially mediated through the restoration of a healthier gut microbial ecosystem (Figure 8).

## 4 Discussion

Dysregulated autophagy and chronic inflammation are hallmarks of IBD pathogenesis; however, the molecular bridges connecting these two processes remain unclear. This study reveals a novel signaling axis—RNF31–TFEB–NLRP3—that critically links ubiquitin‐dependent protein degradation to autophagy dysfunction, intestinal barrier impairment, and inflammation in IBD. Furthermore, we identified the curcumin analog CM‐C1 as an effective multimodal therapeutic agent that targets this axis while also remodeling the gut microbiota, offering a promising interventional strategy.

Our findings solidify the pivotal role of RNF31 in IBD and extend its known functions. While earlier studies established RNF31 as a positive regulator of inflammation by stabilizing the NLRP3 inflammasome (Wang et al. 2023), its upstream mechanisms and other key substrates in the gut epithelium were not fully defined. Our systematic screening identified TFEB, a master transcriptional regulator of autophagy and lysosomal biogenesis, as a novel and functionally critical interaction partner of RNF31. The clinical relevance of this interaction is underscored by the inverse correlation between RNF31 and TFEB protein levels in clinical (Figure 1C,D) and the enrichment of IBD‐related inflammatory pathways associated with low TFEB activity (Figure 1E). Thus, the RNF31–TFEB axis emerges as a central node integrating proteostasis, autophagy, intestinal barrier integrity, and inflammation.

A key mechanistic insight from our work is the context‐dependent regulation of TFEB by LPS. The effect of LPS on TFEB has been reported with apparent discrepancies; low doses or short‐term treatment can activate TFEB and autophagy (Zhang et al. 2020; Hipolito et al. 2019), while high‐dose, prolonged exposure leads to its inhibition (Martina et al. 2023). Our data resolve this paradox by demonstrating that a high inflammatory load (2.5 μg/mL LPS for 24 h) promotes phosphorylation of TFEB at Ser211 (Figure 2A,B), creating a molecular “tag” that facilitates its recognition by the E3 ligase RNF31. This specific binding culminates in the ubiquitination and proteasomal degradation of TFEB, effectively sequestering it in the cytoplasm (Figure 2C) and preventing the transcription of autophagic and lysosomal genes. Consistent with the established role of autophagy in restraining NLRP3 inflammasome activity (Mehto et al. 2019; Hennig et al. 2021; Jiang et al. 2025), our data indicate that the collapse of TFEB‐mediated autophagic flux removes this brake, leading to NLRP3 hyperactivation and perpetuating intestinal inflammation (Li et al. 2021; Kim et al. 2021).

The therapeutic potential of our findings is exemplified by the efficacy of CM‐C1. As a TFEB activator with superior bioavailability compared to curcumin (He et al. 2023; Jiang et al. 2023), CM‐C1 effectively broke the RNF31‐TFEB interaction. It is noteworthy that CM‐C1 did not significantly alter the total or phosphorylated levels of TFEB nor the LPS‐induced up‐regulation of RNF31. Instead, it likely promotes a conformational change or competitive binding that shields phosphorylated TFEB from RNF31 recognition, thereby facilitating its nuclear translocation (El‐Houjeiri et al. 2019; Curnock et al. 2023). This action restored autophagic flux, suppressed NLRP3 inflammasome activation, and reinforced intestinal barrier integrity both in vitro *and* in vivo (Jiang et al. 2025; Liu, Wang, et al. 2025). Critically, TFEB knockdown completely abrogated the anti‐inflammatory effects of CM‐C1 (Figure 5C,D), confirming that TFEB is an indispensable target for CM‐C1 in counteracting the pro‐inflammatory effects of LPS. In the DSS‐induced murine colitis model, these mechanisms translated into improved disease activity index, preserved intestinal mucosal architecture, increased autophagosome numbers in the colon (Figures 6E–H), and reduced systemic levels of pro‐inflammatory cytokines (Figure S3E), further validating the therapeutic potential of CM‐C1.

Our findings are consistent with a growing body of literature linking TFEB‐mediated autophagy to NLRP3 inflammasome suppression. In a diabetic encephalopathy model, TFEB overexpression enhanced autophagic degradation of NLRP3 and alleviated neuroinflammation (Lin et al. 2024). Similarly, in renal epithelial cells, LPS stimulation was shown to inhibit TFEB‐mediated autophagic flux, thereby promoting NLRP3 inflammasome activation (Song et al. 2023). These studies, together with our observations, reinforce the concept that the TFEB‐autophagy axis serves as a key checkpoint for NLRP3 inflammasome activity. Our data extend this paradigm by identifying RNF31 as an upstream E3 ligase that targets TFEB for degradation under inflammatory conditions, thereby linking RNF31‐mediated TFEB suppression to NLRP3 inflammasome hyperactivation.

Beyond its direct impact on host cell signaling, CM‐C1 demonstrated a remarkable ability to remodel the gut microbiota, a key contributor to IBD pathogenesis. The dysbiosis observed in DSS‐treated mice was significantly ameliorated by CM‐C1, which enriched for beneficial taxa like *Eubacterium* (Liu, Wu, et al. 2025), known for its anti‐inflammatory properties through the production of short‐chain fatty acids (Danne et al. 2024; Cai et al. 2022; Fawad et al. 2022; Cong et al. 2023). This microbial remodeling likely synergizes with CM‐C1's direct effects on the RNF31‐TFEB axis: the improved host intestinal environment (restored barrier function, reduced inflammation) fosters a beneficial microbiota, which in turn reinforces gut homeostasis, creating a positive feedback loop that contributes to the alleviation of colitis.

More importantly, Baf A1 completely reversed the CM‐C1‐induced reduction of NLRP3 protein levels, leading to re‐accumulation of NLRP3 (Figure S2C,D). This result demonstrates that the inhibitory effect of CM‐C1 on NLRP3 inflammasome activation is critically dependent on intact autophagic degradation. Collectively, these findings establish that the RNF31‐TFEB axis governs an autophagic checkpoint that directly limits NLRP3 inflammasome activity, and that CM‐C1 disrupts the RNF31‐TFEB interaction to restore this checkpoint, thereby alleviating intestinal inflammation.

## 5 Conclusion

In summary, this study elucidates a novel mechanism in IBD: LPS‐induced phosphorylation of TFEB drives its binding to RNF31 (at TFEB‐S281/T276 and RNF31‐K908), triggering TFEB ubiquitination and proteasomal degradation. This process impedes TFEB nuclear import, suppresses autophagy, and thereby activates the NLRP3 inflammasome to propagate intestinal inflammation. Importantly, we identify the curcumin derivative CM‐C1 as a targeted agent that disrupts the RNF31‐TFEB interaction. By promoting TFEB nuclear localization, CM‐C1 restores autophagy and reverses LPS‐induced pro‐inflammatory effects. Our mechanistic delineation of the RNF31–TFEB–NLRP3 axis, coupled with the validation of CM‐C1's efficacy, provides a crucial foundation for overcoming clinical challenges in IBD and developing novel targeted therapies.

## Author Contributions

**Chunyan Zeng:** validation, writing – review and editing. **Lu Han:** writing – original draft, funding acquisition, methodology, conceptualization, investigation, data curation, writing – review and editing. **Yang Xie:** formal analysis, methodology. **Youxiang Chen:** writing – review and editing, supervision, project administration.

## Funding

This work was supported by the National Natural Science Foundation of China (82360112), the Jiangxi Provincial Natural Science Foundation (20232BAB216018), and the Jiangxi Provincial Traditional Chinese Medicine Administration Science and Technology Plan Project (2024A0032).

## Ethics Statement

All animal experiments were approved by the Institutional Animal Care and Use Committee (IACUC) of The First Affiliated Hospital, Jiangxi Medical College, Nanchang University (Approval No. CDYFY‐IACUC‐202503QR002) and were performed in accordance with relevant guidelines and regulations.

## Conflicts of Interest

The authors declare no conflicts of interest.

## Supporting information


**Figure S1:** Effects of LPS and CM‐C1 on TFEB localization, gene expression, and cell viability in NCM460 cells.
**Figure S2:** CM‐C1 restores autophagic flux and promotes autophagic degradation.
**Figure S3:** CM‐C1 ameliorates gut barrier impairment and systemic inflammation in DSS‐induced colitis.
**Figure S4:** Validation of RNF31 knockdown in vitro and analysis of protein expression in vivo.
**Table S1:** siRNA sequences for human RNF31 and TFEB.
**Table S2:** Details of the antibodies used in the study.
**Table S3:** Sequences of primers for quantitative real‐time PCR.
**Table S4:** Details of the Assay kits used in the study.
**Table S5:** Ubiquitin ligases targeting RNF31 and intersection results across analyses.

## Data Availability

The datasets generated and/or analyzed during the current study are publicly available in the Mendeley Data repository and can be accessed via the following persistent link: https://data.mendeley.com/preview/2379w572tw?a=52f4f342‐7b11‐45da‐8a8b‐3d7c40f3f4f0.
