# Supplementary material for: Targeting the RNF31–TFEB–NLRP3 Axis With a Curcumin Analog to Restore Autophagy and Alleviate Intestinal Inflammation
Source: Food Sci Nutr. 2026 May 23;14(5):e71916. doi: 10.1002/fsn3.71916 (PMC13239829; doi:10.1002/fsn3.71916)
Supplement: Supplementary file 1 — Figure S1: Effects of LPS and CM‐C1 on TFEB localization, gene expression, and cell viability in NCM460 cells. Figure S2: CM‐C1 restores autophagic flux and promotes autophagic degradation. Figure S3: CM‐C1 ameliorates gut barrier impairment and systemic inflammation in DSS‐induced colitis. Figure S4: Validation of RNF31 knockdown in vitro and analysis of protein expression in vivo. Table S1: siRNA sequences for human RNF31 and TFEB. Table S2: Details of the antibodies used in the study. Table S3: Sequences of primers for quantitative real‐time PCR. Table S4: Details of the Assay kits used in the study. Table S5: Ubiquitin ligases targeting RNF31 and intersection results across analyses. [file FSN3-14-e71916-s001.zip › fsn371916-sup-0001-DataS1.docx]

**Supplementary Materials for Targeting the RNF31-TFEB-NLRP3 Axis with a Curcumin Analog to Restore Autophagy and Alleviate Intestinal Inflammation**

Lu Han^1, 2, #^, Yang Xie^1,^ ^4, #^, Chunyan Zeng^3, *^, Youxiang Chen^1, *^.

1 Department of Gastroenterology, Jiangxi Provincial Key Laboratory of Digestive Diseases, Jiangxi Clinical Research Center for Gastroenterology, Digestive Disease Hospital The First Affiliated Hospital, Jiangxi Medical College, Nanchang University, Nanchang, Jiangxi, China.

2 Postdoctoral Research Station, The First Affiliated Hospital, Jiangxi Medical College, Nanchang University, Nanchang, 330006, People's Republic of China.

3 Department of Gastroenterology, Jiangxi Province Hospital of Integrated Chinese and Western Medicine, Nanchang, Jiangxi, China.

4 Digestive Diseases Center, Guangdong Provincial Key Laboratory of Digestive Cancer Research, The Seventh Affiliated Hospital, Sun Yat-Sen University, Shenzhen, Guangdong, 518107, People's Republic of China.

**#** **First author:** Lu Han and Yang Xie contributed equally to this work.

*** Corresponding Author Information:**

Dr Chunyan Zeng, Department of Gastroenterology, Jiangxi Province Hospital of Integrated Chinese and Western Medicine, 90 Bayi Road, Xihu District, Nanchang, 330003, Jiangxi Province China. Email: zengchunyan2024@163.com. Tel: 13767061840.

Dr Youxiang Chen, Department of Gastroenterology, Digestive Disease Hospital, the First Affiliated Hospital of Nanchang University, 17 Yongwaizheng Street, Nanchang, 330006, Jiangxi, China. Email: chenyx102@ncu.edu.cn. Tel: 13879169980.

**This file includes:**

Materials and Methods

Supplementary Figures. S1 to S4

Supplementary Tables. S1 to S4

**Supplementary Figures and Figure Legends**

**
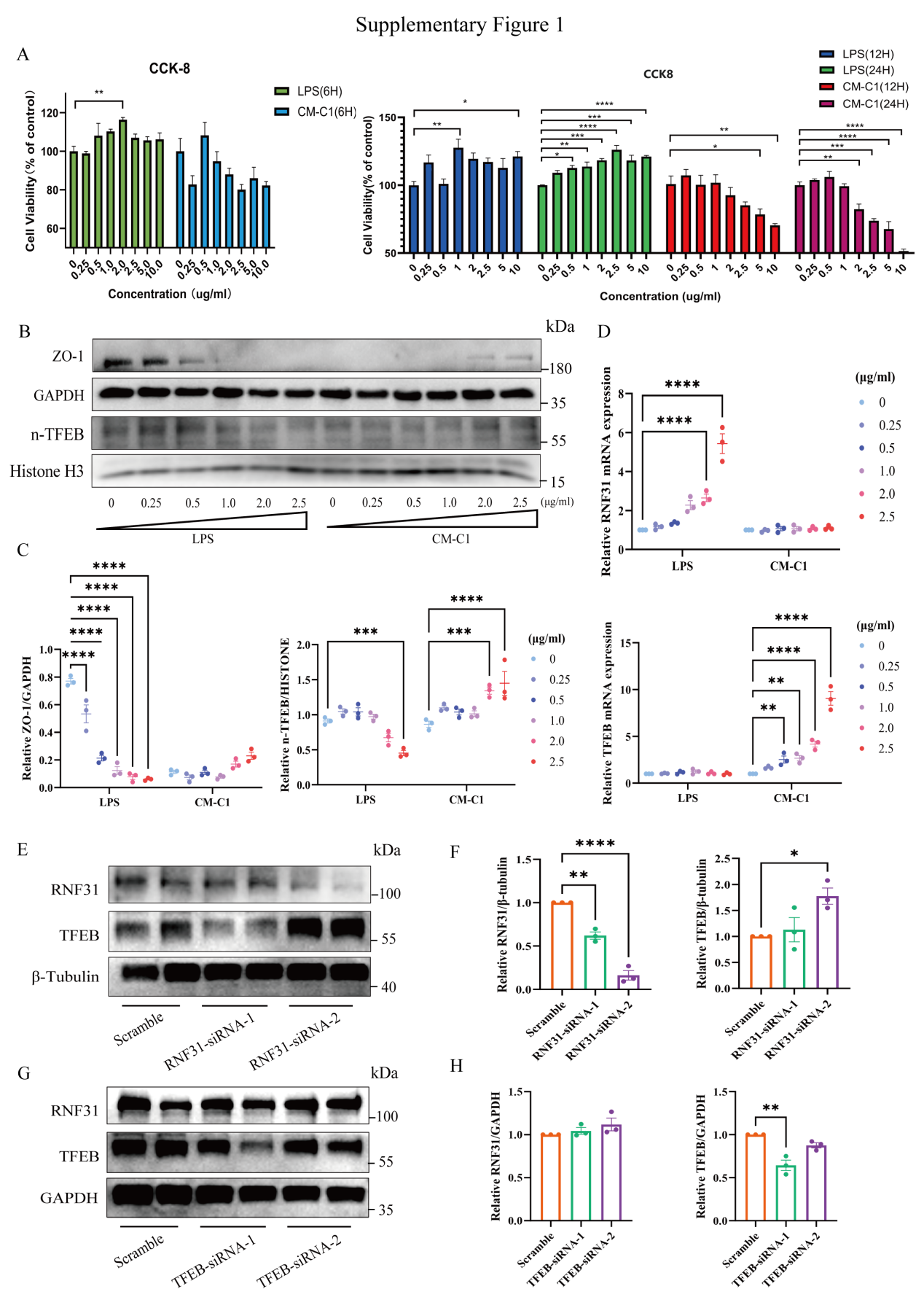
**

**Supplementary Figure 1. Effects of LPS and CM‑C1 on TFEB localization, gene expression, and cell viability in NCM460 cells.**

(A) Cell viability assessed by the CCK‑8 assay in cells treated with a range of concentrations of LPS or CM‑C1 (0–10.0 μg/mL) for 6, 12, and 24 hours.

**(B, C)** (B) Representative immunofluorescence images showing nuclear translocation of TFEB (red) in NCM460 cells treated with LPS or CM‑C1 for 24 h. Nuclei were stained with DAPI (blue). (C) Quantification of nuclear TFEB fluorescence intensity from (B).

1. mRNA expression levels of TFEB and RNF31 determined by qRT‑PCR in cells treated with LPS or CM‑C1 (0–2.5 μg/mL) for 24 h.

**(E, F)** (E) Western blot analysis confirming RNF31 knockdown efficiency in NCM460 cells transfected with control (Scramble) or RNF31‑targeting siRNA (RNF31‑siRNA). (F) Quantification of TFEB and RNF31 protein levels from (E).

**(G, H)** (G) Western blot analysis confirming TFEB knockdown efficiency in NCM460 cells transfected with control (Scramble) or TFEB‑targeting siRNA (TFEB‑siRNA). (H) Quantification of TFEB and RNF31 protein levels from (G).

Data are presented as mean ± SEM from three independent biological experiments (n = 3). Statistical significance was determined by one‑way ANOVA. (**p* < 0.05, ** *p* <0.01, *** *p* <0.001, *****p* < 0.0001).


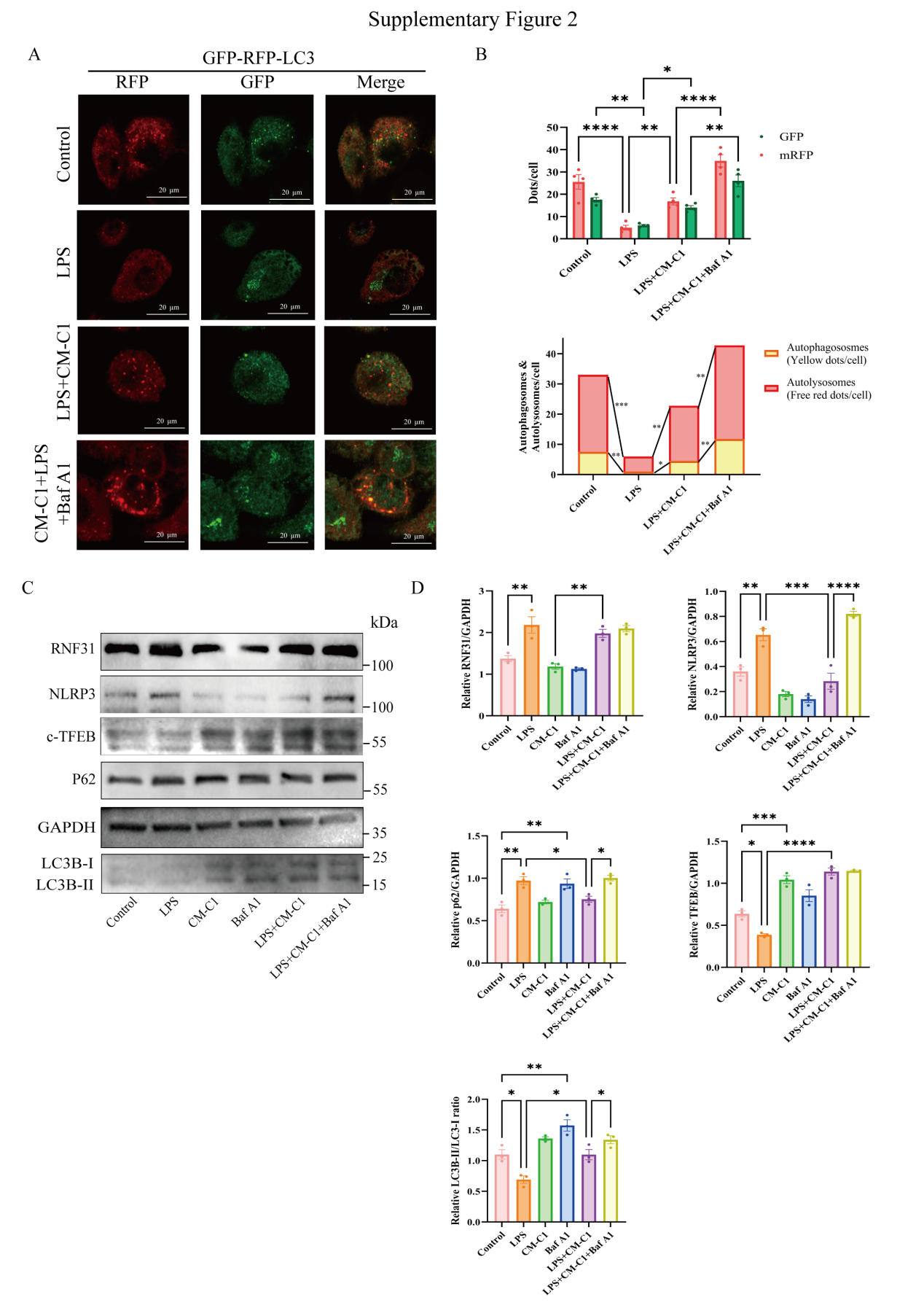


**Supplementary Figure 2. CM‑C1 restores autophagic flux and promotes autophagic degradation.**

**(A, B)** (A) Representative images of GFP‑RFP‑LC3 assays in NCM460 cells stimulated with LPS (2.5 μg/mL) for 24 h, with or without CM‑C1 (2.0 μg/mL). To evaluate the effect of CM‑C1 on autophagic flux, cells were additionally treated with Bafilomycin A1 (Baf A1, 100 nM) for 8 h. (B) Left: Quantification of GFP and mRFP puncta per cell from (A). Right: Numbers of autophagosomes and autolysosomes per cell in each group.

**(C, D)** (C) Western blot analysis confirming the autophagic effect of CM‑C1 in NCM460 cells under LPS stimulation, with or without Baf A1. (D) Quantification of TFEB and RNF31 protein levels from (C).

Data are presented as mean ± SEM from three independent biological experiments (n = 3). Statistical significance was determined by one‑way ANOVA. (**p* < 0.05, ** *p* <0.01, *** *p* <0.001, *****p* < 0.0001).

**
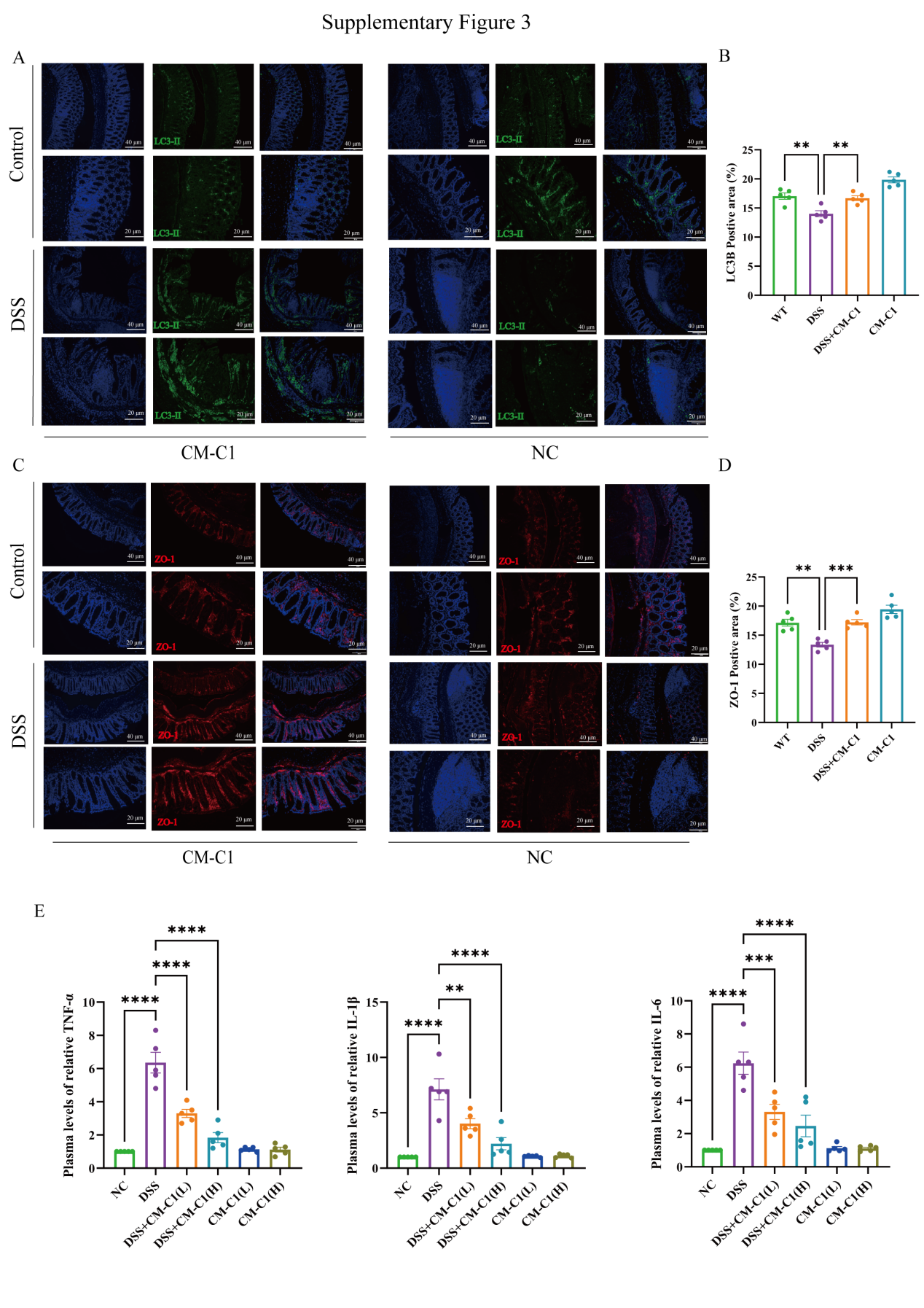
**

**Supplementary Figure 3. CM-C1 ameliorates gut barrier impairment and systemic inflammation in DSS-induced colitis.**

1. **D)** (A, C) Representative immunofluorescence (IF) images of LC3B (A) and ZO‑1 (C) in colon tissues from WT mice treated with or without DSS and CM‑C1. (B, D) Corresponding quantification of fluorescence intensity for LC3B (B) and ZO‑1 (D).
2. Plasma levels of TNF-α, IL-6, and IL-1β in each group.

Data are presented as mean ± SEM (n = 5 mice per group). Statistical significance was determined by one-way ANOVA (**p* < 0.05, ***p* < 0.01, ****p* < 0.001, *****p* < 0.0001).


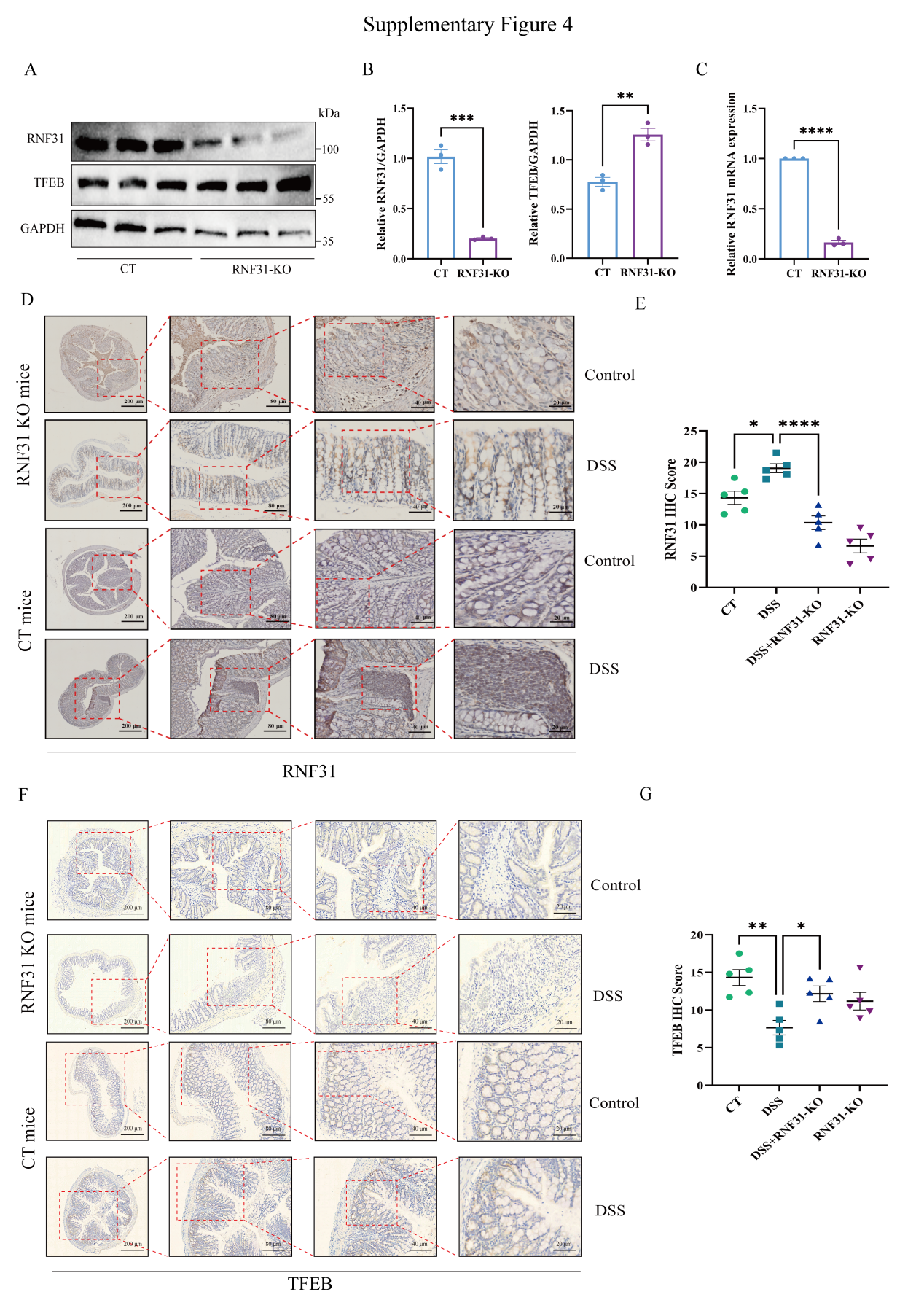


**Supplementary Figure 4. Validation of RNF31 knockdown *in vitro* and analysis of protein expression *in vivo*.**

**(A, B)** (A) Western blot analysis confirming knockdown efficiency in intestinal epithelial‑specific RNF31 knockout (RNF31‑KO) mice. (B) Quantification of RNF31 protein levels from (A).

**(C)** Relative mRNA expression of RNF31 in colon tissues from RNF31‑KO mice.

**(D-G)** Representative immunohistochemistry (IHC) images and quantification of RNF31 and TFEB in colon tissues from RNF31^fl/fl^ (CT) and RNF31‑KO mice, with or without DSS treatment. (D) IHC images of RNF31; (E) quantification of RNF31. (F) IHC images of TFEB; (G) quantification of TFEB.

**
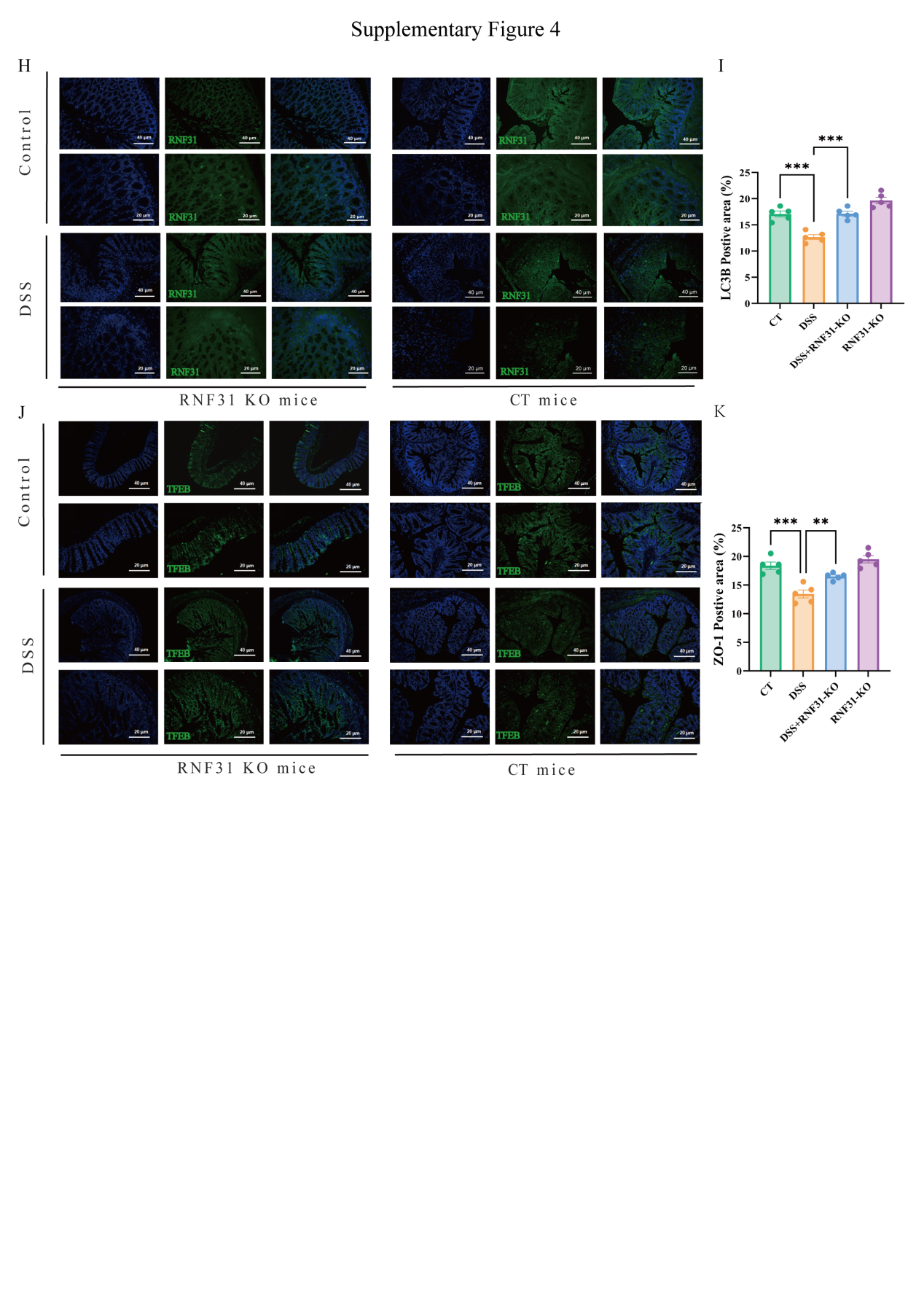
**

**(H-K)** (H, J) Representative IF images of LC3B (H) and ZO‑1 (J) in colon sections from RNF31‑KO and CT mice treated with DSS, with or without CM‑C1. (I, K) Corresponding quantification of fluorescence intensity.

Data are mean ± SEM. (B, C) n = 3 per group from three independent experiments; (E, G) n = 5 mice per group. Statistical significance was determined by Student's t‑test for (B) and (C), and by one‑way ANOVA for (I) and (K). (**p* < 0.05, ***p* < 0.01, ****p* < 0.001, *****p* < 0.0001).

**Supplementary Tables:**

**Supplementary Table 1. siRNA sequences for human RNF31 and TFEB.**

si-RNA sequences designed by oligobio Biotechnology, Co, Ltd (China)

Sequence (5′ → 3′)

Human RNF31-siRNA-1

5′-CCUAUGCGUUGUUCCAUAATT-3′

Human RNF31-siRNA-2

5′-UUAUGGAACAACGCAUAGGTT-3′

Human TFEB-siRNA-1

5′-GCAAGGUCAAGAUCAAGAATT-3′

Human TFEB-siRNA-2

5′-GGAACUGACUCUCAAGAAUTT-3′

**Supplementary Table 2. Details of the antibodies used in the study.**

| **Antibody names** | **Brand** | **Product code** | **Application** |
| --- | --- | --- | --- |
| Anti-RNF31/HOIP | Abcam | AB315162 | WB, 1:1000  IF, 1:200  IP, 1:100 |
| Anti-RNF31/HOIP | Abcam | AB187976 | IHC, 1:200 |
| Anti-TFEB | Zenbio | 13372-1-AP | WB, 1:1000  IF, 1:200  IP, 1:100  IHC, 1:200 |
| Anti-NLRP3 | Proteintech | 30109-1-AP | WB, 1:1000 |
| Anti-ZO-1 | ThermoFisher | 61-7300 | WB, 1:1000  IF, 1:200 |
| Anti-Caspase-1 | Proteintech | 22915-1-AP | WB, 1:1000 |
| Anti-IL-1β | Proteintech | 16806-1-AP | WB, 1:1000 |
| Anti-SQSTM1/p62 | Zenbio | 380612 | WB, 1:1000 |
| Anti-CYLD | Zenbio | 618251 | WB, 1:1000 |
| Anti-p-TFEB (Ser211) | Cell Signaling Technology | 37681 | WB: 1:1000 |
| Anti-LC3B | Proteintech | 14600-1-AP | IF, 1:150  WB, 1:1000 |
| Anti-IL-18 | Proteintech | 10663-1-AP | WB, 1:1000 |
| Anti-β-Tubulin | Cell Signaling Technology | 2146S | WB, 1:1000 |
| Anti-GAPDH | Cell Signaling Technology | 14C10 | WB, 1:3000 |
| Anti-Histone H3 | Zenbio | R24572 | WB, 1:1000 |
| Goat anti-Rabbit IgG | Proteintech | B900210 | WB, 1:10000 |
| Alexa Fluor®488 Goat anti-rabbit IgG (H+L) | Abcam | ab150077 | IF, 1:500 |
| Goat Anti-mouse IgG (H+L) | Boster Biological Technology | BA1051 | WB, 1:10000 |
| Goat Anti-rabbit IgG (H+L) | Boster Biological Technology | AB_2927669 | WB, 1:10000 |
| Alexa Fluor®647 Goat anti-rabbit IgG (H+L) | Abcam | ab150079 | IF, 1:500 |
| Anti-HA | MedChemExpress | YA4625 | WB, 1:1000 |
| Anti-FLAG | MedChemExpress | YA780 | WB, 1:1000 |
| Anti-His | MedChemExpress | YA4624 | WB, 1:1000 |

**Supplementary Table 3. Sequences of primers for quantitative real-time PCR.**

| **Genes** | **Primer sequences (5’ → 3’)** |
| --- | --- |
| Hum-RNF31-F | GAGCCCCGAAACTACCTCAAC |
| Hum-RNF31-R | CTTGACACCACGCCAGTACC |
| Hum-GAPDH-F | GCACCGTCAAGGCTGAGAAC |
| Hum-GAPDH-R | TGGTGAAGACGCCAGTGGA |
| Hum-TFEB-F | CAGCAGTCGCAGCATCAGAAGG |
| Hum-TFEB-R | TGTTGCCAGCGGAGGAGGAC |
| Hum-IL-6-F | ACTCACCTCTTCAGAACGAATTG |
| Hum-IL-6-R | CCATCTTTGGAAGGTTCAGGTTG |
| Hum-TNF-α-F | CCTCTCTCTAATCAGCCCTCTG |
| Hum-TNF-α-R | GAGGACCTGGGAGTAGATGAG |
| Hum-IL-18-F | CTTTGGCTGCCATGTCAGAAG |
| Hum-IL-18-R | TCTGACATGGCAGCCATTGT |
| Mus-RNF31-F | GGAAGCGTCTCATCAGCATC |
| Mus-RNF31-R | TCCAGGTAGGCGTTGTCATT |
| Mus-TFEB-F | CAGCGAGTACATCACCAACAG |
| Mus-TFEB-R | GCACACATCCACACCAATCTC |
| Mus-GAPDH-F | AGGTCGGTGTGAACGGATTTG |
| Mus-GAPDH-R | TGTAGACCATGTAGTTGAGGTCA |
| Mus-IL-6-F | TAGTCCTTCCTACCCCAATTTCC |
| Mus-IL-6-R | TTGGTCCTTAGCCACTCCTTC |
| Mus-TNF-α-F | CCCTCACACTCAGATCATCTTCT |
| Mus-TNF-α-R | GCTACGACGTGGGCTACAG |
| Mus-IL-18-F | GACTCTTGCGTCAACTTCAAGG |
| Mus-IL-18-R | CAGGCTGTCTTTTGTCAACGA |

**Supplementary Table 4. Details of the Assay kits used in the study.**

| **Assay kits used in the study** | **Brand** | **Product code** | **Standard** |
| --- | --- | --- | --- |
| IL-6 | Boster Biological Technology | EK0410 | 100T/48S |
| TNF-α | Boster Biological Technology | EK0392 | 50T/48S |
| IL-1β | Boster Biological Technology | EK0525 | 96T |
